# Anticancer, antimicrobial and molecular docking analysis of newly synthesized iodoquinazoline derivatives

**DOI:** 10.1186/s13568-025-01899-1

**Published:** 2025-06-18

**Authors:** Khaled M. Aboshanab, Amr S. Bishr, Siti Azma Jusoh, Mohammad Y. Alshahrani, Khondaker Miraz Rahman, Ahmed M. Alafeefy

**Affiliations:** 1 Department of Microbiology and Immunology, Faculty of Pharmacy, Ain Shams University, Cairo, 11566 Egypt; 2 Department of Pharmacology and Life Sciences, Faculty of Pharmacy, Universiti Teknologi MARA (UiTM), Puncak Alam Campus, 42300 Bandar Puncak Alam, Selangor Malaysia; 3 Institute of Pathology, Laboratory, and Forensic Medicine (I-PPerForM), Universiti Teknologi MARA, 47000 Sungai Buloh, Selangor Malaysia; 4 Central Labs, King Khalid University, P.O. Box 960, AlQura’a, Abha, Saudi Arabia; 5 Department of Clinical Laboratory Sciences, College of Applied Medical Sciences, King Khalid University, P.O. Box 61413, 9088 Abha, Saudi Arabia; 6 School of Cancer and Pharmaceutical Science, King’s College London, 150 Stamford Street, London, SE1 9NH UK; 7 Department of Pharmaceutical Chemistry, Faculty of Pharmacy, Universiti Teknologi MARA (UiTM), Puncak Alam Campus, 42300 Bandar Puncak Alam, Selangor Malaysia

**Keywords:** Quinazoline, Sulfonamide, Carbonic anhydrase, Cancer, Antimicrobial, Molecular docking

## Abstract

The 4-anilinoquinazoline sulfonamide derivatives in medicinal chemistry are well-known antitumor backbones via different mechanisms. Herein, we reported the synthesis of five new 2,4-disubstituted-6-iodoquinazoline derivatives coded compounds 3a–e. The respective compounds were examined for their antiproliferative, antimicrobial, and carbonic anhydrase XII (CAXII) inhibitory activity against four cancerous cell lines. Compound 3c was proven to be the most effective as an anticancer. It stopped the growth of the four used tumor cell lines at concentrations ranging from 4.0 to 8.0 µM as compared to the reference doxorubicin (2.3–3.25 µM). Compound 3b, was able to stop the proliferation of the tumor cell lines with IC_50_ between 6.0 and 9.0 µM. The five compounds exhibited both broad-spectrum and antifungal action; however, their antibacterial activities against Gram-positive bacteria were superior to those of Gram-negative. Compound 3c showed the strongest antibacterial and antifungal activities, followed by compound 3b. In conclusion, the 4-substituted-anilino derivatives equipped with sulfonamide at position 4 of the quinazoline nucleus are suitable antitumor lead compounds. Moreover, the 4-methoxyphenyl substituent at position-2 has a better impact on activity than unsubstituted or even other substituents. The lab results are aligned with molecular docking analysis of the respective compounds against the potential targets, including CAXII, human thymidylate synthase (hTS), and human thymidine kinase (hTK), for the anticancer activities and DHFR of *E. coli* and *S. aureus* for the antibacterial properties. Compounds 3c and 3b are highly recommended for preclinical and clinical evaluation as anticancer and/or antibacterial agents for potential use in humans.

**Supplementary Information:**

The online version contains supplementary material available at 10.1186/s13568-025-01899-1.

## Introduction

Globally, the medical profession is concerned about cancer as a major health issue. Developing nations will see a higher increase in cases than industrialized ones. By that time, cancer may overtake all other causes of death worldwide (Sung et al. 2021). Over 70% of cancer-related deaths worldwide take place in low-resource nations, where most cases are discovered too late to get appropriate treatment (Yu et al. 2024). There has been significant advancement in several areas of cancer research, but none as notable as the discovery of penicillin, which is used in antibacterial chemotherapy (Maestri et al. 2017). After heart disease, cancer is the second most common cause of mortality in the Western world (Szewc et al. 2022). Whereas mortality for less deadly cancers may represent not just the cancer risk but also the stage of the disease at diagnosis, the effectiveness of treatment, and the prognosis after treatment, mortality for more deadly cancers roughly corresponds to incidence (Jamroskovic et al. 2020).

Moreover, one of the most pressing issues in public health that needs immediate attention is antibiotic resistance and limited therapeutic options for infections caused by clinically relevant pathogens (Miller-Petrie et al. 2017; El-Sayed et al. 2020; Miethke et al. 2021; Tang et al. 2023). The World Health Organization (WHO) estimates that in 2019, 1.27 million deaths were directly linked to antimicrobial medication resistance (World Health Organization, WHO 2023). It is estimated that by 2050, there will be 10 million deaths worldwide, with this potentially being the main cause of mortality that year (WHO 2023). Antibiotic resistance also presents a significant economic risk, according to World Bank research titled "Drug-resistant infections: a threat to our economic future (Miller-Petrie et al. 2017; Tang et al. 2023). Among the many strategies to address this challenge are innovation and finding new molecules that can be used to combat antibiotic resistance and treat infections caused by clinically relevant pathogens (Abdelaziz et al. 2021; Banoub et al. 2021).

On the other hand, from a chemistry point of view, quinazoline derivatives have been reported to exhibit anticancer properties against multiple cancer cell types (Chang et al. 2020). Interestingly, these agents have shown inhibitory activity against human thymidylate synthase (hTS), thymidine kinase (hTK), DNA repair, and dihydrofolate reductase (DHFR) (El-Sayed et al. 2019; Das et al. 2021; Angeli 2024; Chen et al. 2024). Further, sulfonamide derivatives are famous for their antitumor activity due to their ability to inhibit carbonic anhydrase (CA) isoforms, and a wide range of such compounds have been used in various medicinal chemistry applications (Supuran 2012; Alafeefy et al. 2016; Amir et al. 2022). In addition, sulfonamides have been shown to accumulate in hypoxic tumors (Supuran 2024) selectively. Therefore, combining sulfonamides with quinazoline derivatives in one molecule is expected to enhance antitumor and antimicrobial activity and reduce toxicity. To achieve this goal, we adopted documented chemical procedures. So, based on previous results in our hands, herein, we report the synthesis of five novel di-substituted 4-anilinoquinazoline derivatives, followed by testing their ability to inhibit CAXII and evaluating their antiproliferative as well as their antimicrobial properties. Moreover, docking analysis of the respective compounds against possible targets was conducted to elucidate their potential mechanism of action.

## Materials and methods

### Chemistry

Melting points (m.p.) were measured in open capillaries using an uncorrected Gallenkamp melting point apparatus (Sanyo Gallenkamp, Southborough, UK). Using a PerkinElmer FT-IR (Spectrum BX) spectrophotometer, the infrared (IR) spectra were recorded on KBr discs (vmax in cm^−1^). A BRUKER Resonance spectrometer (850 MHz “megahertz”) was used to record the 1H- and 13C NMR spectra. In Hertz (Hz), coupling constants are expressed. The DMSO-*d*_*6*_ solvent was purchased from Goss Scientific Instruments and was kept in silica gel desiccators. The solvent peak serves as the internal standard for expressing chemical shifts in *δ* values (ppm); the multiplicity of resonance peaks is denoted by the symbols singlet (s), doublet (d), triplet (t), quartet (q), and multiplet (m) as previously reported (El-Sayed et al. 2019).

### Spectral information

The compounds' structures were validated by IR, NMR, and elemental analysis. Liquid Chromatography-Mass Spectrometry (LC–MS) determined the compounds' purity (˃ 95%), with results falling within 0.4% of the computed values. By matching their analytical and physicochemical data with previously published data, all known chemicals were identified. Using conventional methods, all solvents and reagents were dried and purified (Alafeefy et al. 2016; El-Sayed et al. 2019).

### Synthesis of 4-chloroquinazolines 2a–d

Portion-wise 2-substituted-quinazolin-4(3H)-one 1a–d (3.0 g) was added to a magnetically agitated solution of phosphorus oxychloride (20 mL) and N,N-dimethylaniline (1 mL) at 0 °C. For eight hours, the reaction mixture was refluxed. After adding the latter to freezing water, 2 N NaOH was used to bring the pH down to an alkaline level. Dichloromethane was used to extract the reaction mixture. After the organic extracts were filtered and dried over Na_2_SO_4_, the solvent was extracted using distillation. After recrystallizing the resulting solid from ethanol, ^9–11^ was produced (Alafeefy et al. 2016; El-Sayed et al. 2019; Angeli 2024).

### Synthesis of compounds 3a–e

A solution of sulfanilamide or sulfathiazole (1 mmol) in ethanol was added to a stirred solution of 4-chloro-6-iodoquinazolines 2a–d (1 mmol) in refluxing ethanol (15 mL). For three hours, the reaction mixture was refluxed. Compounds 3a to 3e were obtained by filtering the precipitate that had developed and crystallizing it from ethanol.

**6-Iodo-4-(4-aminosulphonylphenyl-amino) quinazoline (3a).** Yield (73%); m.p. > 300 °C; IR ν 3389, 3367, 3235 (NH, NH_2_), 3204 (CH arom.), 1387, 1221 (SO_2_), cm^1^; ^1^H NMR (DSMO-*d*_*6*_) *δ* 7.34 (brs, 2H, NH_2_, D_2_O exchange.), 7.51–7.63 (m, 2H, Ar-H), 7.94–7.97 (m, 2H, Ar-H), 8.05–8.15 (m, 1H, Ar-H), 8.38–8.41 (m, 2H, Ar-H), 8.79–8.81 (m, 2H, Ar-H), 11.05 (s, 1H, NH, D_2_O, exchange); ^13^C NMR (DSMO-*d*_*6*_) *δ* 94.4, 114.1, 120.3, 125.0, 125.2, 126.4, 128.7, 136.4, 138.5, 139.2, 141.5, 151.1, 160.5; MS m/z (Rel. Int.). Anal. (C_14_H_11_IN_4_O_2_S, 300.07). C, 55.99 (56.26); H, 4.03 (4.28); N, 18.66 (18.43); S, 10.67 (10.83); MS m/z (Rel. Int.) 425, (M^+^, 425). Anal. (C_14_H_11_IN_4_O_2_S_,_ 426.27). C, 39.44 (39.45); H, 2.60 (2.62); N, 13.15 (13.16); S, 7.53(7.55).

**6-Iodo-4-(4-aminosulphonylphenyl-amino)-2-(4-methoxyphenyl)-quinazoline (3b).** Yield (69%); m.p. > 300 °C; IR ν 3421, 3381, 3218 (NH, NH_2_), 3125 (CH arom.), 2977, 2873 (CH- aliph.), 1366, 1189 (SO_2_)cm^−1^; ^1^H NMR (DSMO-*d*_*6*_) *δ* 3.76 (s, 3H, OCH_3_), 7.48 (brs, 2H, NH_2_, D_2_O, exchange), 7.76–7.78 (t, 2H, Ar-H), 7.89–7.91 (s, 1H, Ar-H), 8.05–8.15 (m, 4H, Ar-H), 8.32–8.40 (m, 2H, Ar-H), 8.94–8.96 (d, 2H, Ar-H), 11.71 (s, 1H, NH, D_2_O, exchange.); ^13^C NMR (DSMO-d_6_) *δ* 55.4 (OCH_3_), 93.7, 114.8, 120.3, 122.3, 124.7, 124.9, 126.5, 127.9, 132.4, 136.0, 138.7, 141.2, 156.4, 158.9, 163.5, 170.2; MS m/z (Rel. Int.) 532 (M^+^, 120). Anal. (C_21_H_17_IN_4_O_3_S_,_ 532.36). C, 47.38 (47.16); H, 3.22 (2.99); N, 10.52 (10.47); S, 6.02 (5.84).

**6-Iodo-2-phenyl-4-(4-(thiazol-2-yl) aminosulphonylphenyl-amino) quinazoline (3c)**. Yield (71%); m.p. > 300 °C; IR ν 3324, 3247 (2NH), 3121 (CH arom.), 1373, 1180 (SO_2_)cm^−1^; ^1^H NMR (DSMO-d_6_) *δ* 6.82 (s, 1H, Ar-H), 7.27–7.28 (m, 3H, Ar-H), 7.46–7.47 (m, 4H, Ar-H), 7.84–7.85 (d, 1H, Ar-H), 7.93–7.95 (d, 1H, Ar-H), 7.98–8.12 (d, 2H, Ar-H), 8.34–8.36 (d, 2H, Ar-H), 8.93 (brs,1H, NH, D_2_O, exchange.), 11.78 (s, 1H, NH, D_2_O, exchange.); ^13^C NMR (DSMO-*d*_*6*_) *δ* 93.9, 108.3, 112.8, 119.9, 124.2, 124.5, 124.6, 126.5, 128.2, 129.0, 129.2, 133.2, 136.0, 139.2, 140.3, 155.3, 159.1, 159.4, 168.8; MS m/z (Rel. Int.); MS m/z (Rel. Int.) 585 (M^+^, 127). Anal. (C_23_H_16_IN_5_O_2_S_2,_ 585.44). C, 47.19 (46.98); H, 2.75 (2.88); N, 11.96 (12.05); S, 10.95 (11.06).

**6-Iodo-2-(4-methoxyphenyl)-4-(4-(thiazol-2-yl) aminosulphonylphenyl-amino)-quinazoline (3d).** Yield (71%); m.p. > 300 °C; IR ν 3411, 3383, 3228 (NH, NH_2_), 3121 (CH arom.), 2978, 2871 (CH aliph.), 1372, 1185 (SO_2_) cm^−1^; 1 H NMR (DSMO-d_6_) 3.74 (s, 3H, OCH_3_), 7.45 (brs, 2H, NH_2_, D_2_O, exchange.), 7.76–7.80 (t, 2H, Ar-H), 7.97–7.99 (d, 2H, Ar-H), 8.06–8.14 (m, 4H, Ar-H), 8.33–8.39 (m, 2H, Ar-H), 8.91–8.93 (d, 2H, Ar-H), 11.74 (s, 1H, NH, D_2_O, exchange.); ^13^C NMR (DSMO-d6) d 55.7 (OCH_3_), 93.2, 112.6, 114.3, 120.5, 121.5, 124.1, 124.8, 126.1, 127.9, 131.6, 136.2, 138.7, 141.4, 155.9, 157.6, 164.1, 169.4; MS m/z (Rel. Int.) Anal. (C_24_H_18_IN_5_O_3_S_2,_ 615.46). C, 46.84 (64.72); H, 2.95 (3.07); N, 7.80 (7.69); S, 10.42 (10.35).

**6-Iodo-2-(3,4-dimethoxyphenyl)-4-(4-(thiazol-2-yl) aminosulphonylphenyl-amino)-quinazoline (3e).** Yield (57%); m.p. > 300 °C; IR ν 3337, 3252 (2NH), 3133 (CH arom.), 2977, 2892 (CH aliph.), 1385,1190 (SO_2_) cm^−1^; ^1^H NMR (DSMO-d_6_) *δ* 3.78(s, 6H, 2OCH_3_), 6.79(s, 1H, Ar-H), 7.14–7.15 (d, 2H, Ar-H), 7.28–7.30 (d, 2H, Ar-H), 7.75–7.95 (m, 4H, Ar-H), 8.02–8.06 (m, 3H, Ar-H), 8.85 (brs, 1H, NH, D_2_O, exchange.), 11.70 (brs, 1H, NH, D_2_O, exchange.); ^13^C NMR (DSMO-d_6_) *δ* 54.7 (OCH_3_), 96.3, 111.4, 112.3, 120.0, 120.7, 124.5, 126.1, 127.9, 128.9, 129.6, 136.2, 140.2, 145.8, 148.7, 151.4, 155.4, 159.4, 167.6,170.3; MS m/z (Rel. Int.) 645 (M^+^, 32); Anal. (C_25_H_20_IN_5_O_4_S_2,_ 645.49). C, 46.52 (46.58); H, 3.12 (3.15); N, 10.85 (10.89); S, 9.93 (9.95).

### Antitumor testing

#### Cell culture general method

Four immortalized human cancer cell lines served as a panel for testing the compounds' cytotoxicity. The STAT3-null A4 cell line (with homozygously deleted STAT3) is a sub-line of the DLD1 colorectal carcinoma cell line and was a gift from Professor David Thurston, Emeritus Professor of Drug Discovery, King’s College London. The American Type Culture Collection and LGC provided the cell lines for HeLa (human cervical cancer), MIA-PaCa-2 (human pancreatic adenocarcinoma), MDA MB231 (triple negative human breast cancer), and NCI H1975 (human non-small cell lung cancer). Every cell line was kept in monolayer culture in 75 cm^2^ flasks (TPP, Switzerland) at 37 °C in a humidified environment containing 5% CO_2_. The HeLa line was kept in Dulbecco's Modified Eagles Media (DMEM; Invitrogen), which was enhanced with L-glutamine (2 mM; Invitrogen), non-essential amino acids (1 x; Invitrogen), 10% v/v fetal bovine serum, and Penicillin–Streptomycin (1% v/v; Invitrogen).

Dulbecco's MEM was utilized for MIA PaCa2, and it was enhanced with fetal calf serum (10%, Biosera UK) and L-glutamine (2 mM; Invitrogen). The following materials were employed for sub-culturing: L-glutamine (2 mM; Invitrogen), fetal bovine serum (10%, Biosera UK), high glucose DMEM (4.5 g/l; Invitrogen), Penicillin–Streptomycin (1% v/v, Invitrogen), and non-essential amino acids (1x; Invitrogen). Penicillin–Streptomycin (1% v/v, Invitrogen) and fetal bovine serum (10% v/v; Invitrogen) were added to Roswell Park Memorial Institute Medium 1640 (RPMI 1640, Invitrogen) to sustain the NCI H1975 cell lines. Cells were passaged by rinsing them in PBS (GIBCO 14040, Invitrogen, UK), culturing them with trypsin (GIBCO 25300, Invitrogen, UK), and then reseeding them into new media. Cells were counted for seeding on a non-adherent suspension of cells that were rinsed in PBS, trypsinized, centrifuged at 8 °C for 5 min at 8000 rpm, and then re-suspended in new media using a Neubauer hemocytometer (Assistant, Germany) by microscopy (Nikon, USA).

### MTT assay

Using the proper medium, the cells were cultured under standard cell culture conditions at 37 °C in a humidified atmosphere with 5% CO_2_. Depending on the cell line, 5,000–20,000 cells were added per well after the cell count was adjusted to 10^5^ cells/mL. After a 24-h incubation period, 1 μL of the suitable inhibitor doses were applied in triplicate to the wells (Freimoser et al. 1999). The MTT colorimetric test (3-(4,5-Dimethylthiazol-2-yl)-2,5-diphenyltetrazolium bromide) (Lancaster Synthesis Ltd, UK) was used to assess the cytotoxicity of each chemical after 96 h of continuous exposure (Freimoser et al. 1999). Using an Envision Plate Reader from PerkinElmer, USA, spectrophotometry was used to measure absorbance at λ = 570 nm. Using the Prism Graphpad Prism® program, a dose–response analysis was used to get the IC50 values. MDA MB231; HeLa; MIA Cell lysis buffer was added to PaCa2 cells after they had been exposed to different doses of chemical 3e. The cells were then collected. Following the electrophoresis of total cell lysates on precast TGX gels, β-actin and CAXII antibodies were immunoblotted (Ghomashi et al. 2023). Doxorubicin was used as a positive control (Ghomashi et al. 2023).

### Evaluation of the carbonic anhydrase XII inhibition

The carbonic anhydrase (CAXII) inhibitory activity of the newly synthesized compounds (3a–e) was carried out on a 96-well flat-bottom plate as previously reported (Ur Rehman et al. 2020). The 200 µL total reaction volume contained 20 µL of test compounds produced in DMSO, 140 µL of HEPES–tris buffer, 20 µL of buffer-processed purified bovine erythrocyte CA-II (0.1 mg/mL), and 20 µL of a 4-nitrophenyl acetate solution (Shank et al. 2005). Acetazolamide was used as a standard CAII inhibitor.

### Evaluation of the antibacterial and antifungal activities

The five newly synthesized compounds were first qualitatively evaluated for antibacterial and antifungal activities using Muller-Hinton agar medium (MHA) through the agar well diffusion technique as previously described (Balouiri et al. 2016). The bacterial test was performed against the following standard strains: *S. aureus* (SA) ATCC 25913, *E. coli* (EC) ATCC 25922, *P. aeruginosa* (PA) ATCC 27853, *K. pneumoniae* (KP) ATCC 49619. The antifungal activity was evaluated against *C. albicans* (CA) ATCC 14053 (CLSI 2021). A bacterial culture and preparation of wells on a seeded MHA plate was performed as previously described (Isleem et al. 2025). The experiment was done in triplicate according to CLSI guidelines (CLSI 2021). Secondly, the compounds that gave positive inhibition zones were selected for MIC determination using broth microdilution assay according to CLSI guidelines (CLSI 2021). Ciprofloxacin and fluconazole were used as positive controls for evaluating the antibacterial and antifungal activities, respectively.

### Computational analyses

#### ADMET prediction

Absorption, distribution, metabolism, excretion, and toxicity (ADMET) prediction of compound 3a–e was performed using the ADME-AI web server (Swanson et al. 2024).

#### Molecular docking

**Fig. 1:**
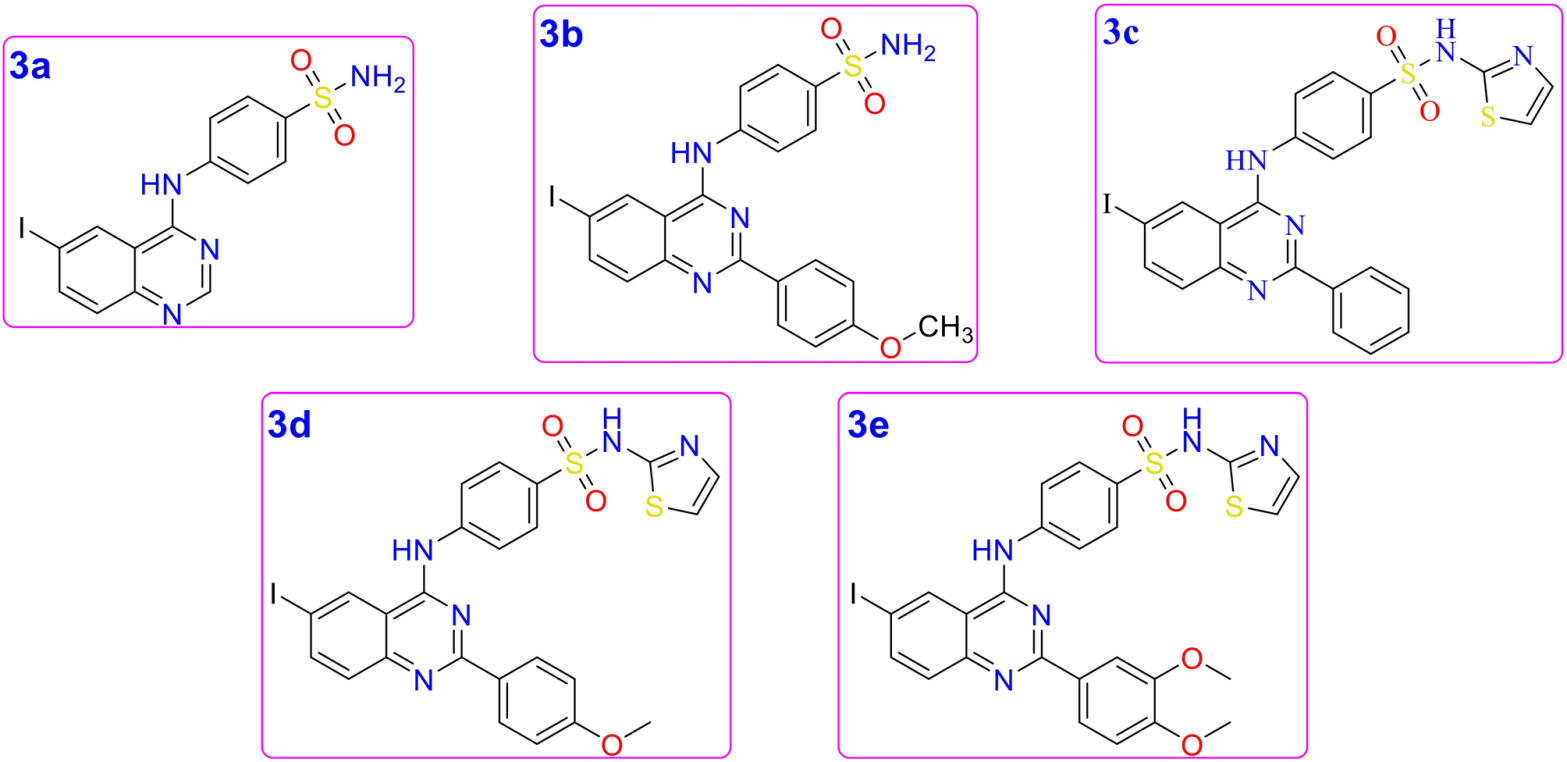
The 2D structures of the 2-substituted-4-anilino-6-iodoquinazoline derivatives (compounds 3a–e)

The initial structures of the compounds were constructed using ChemDraw. The structural information of compounds 3a–e in.cdx format (Fig. 1) was then converted into 3D atomic coordinates (PDB format) using OpenBabel (O’Boyle et al. 2011). The X-ray crystallography structures of the target receptors—CAXII, hTS, hTK, and DHFR of *E. coli* and *S. aureus*—were retrieved from the Protein Data Bank (PDB IDs: 7PUW, 1HVY, 1W4R, 1DDR, 3FRD, respectively). All target receptors are of human origin except for dihydrofolate reductase, derived from *E. coli* (representing Gram-negative) and *S. aureus* (representing Gram-positive) bacterial pathogens. Molecular docking was performed using MolModa, a docking program integrating AutoDock Vina as the docking tool (Eberhardt et al. 2021; Kochnev et al. 2024). MolModa was used to preprocess the PDB files by removing other co-crystallized molecules except the target protein (such as ligand, ions, and water), and its duplicate subunits. The program was also utilized to add hydrogen atoms, optimize protein side chains, and protonate the compounds and proteins at physiological pH (pH 7.4). The binding pocket was identified based on the co-crystallized ligand in the catalytic site of each target protein. The docking exhaustiveness was set to 8, and the box dimension (x, y, z) was set to 20 Å × 20 Å × 20 Å, with the box center coordinates adjusted to center on the co-crystallized ligand. Each docking run was performed three times. The ligand poses with the highest docking scores for each receptor were selected for further analysis. PoseEdit was utilized to analyse interactions (hydrogen bonds, non-polar interactions) between the target protein and ligand (Diedrich et al. 2023).

## Results

### Chemistry

**Fig. 2:**
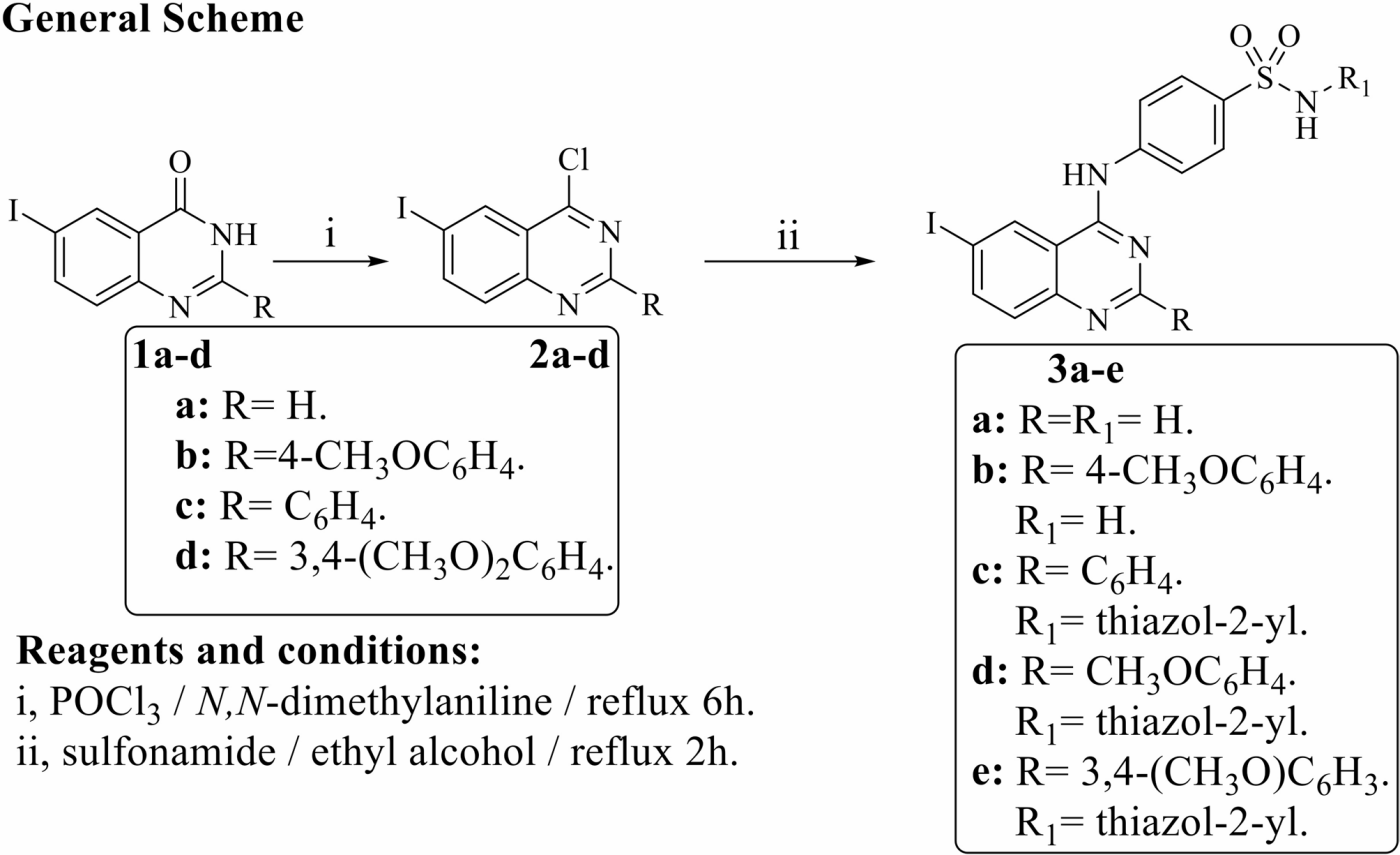
Scheme 1. Chemical synthesis of compounds 3a–e

The target quinazoline derivatives were prepared according to our previously reported procedure (Alafeefy et al. 2016) as depicted in Fig. 2. The key intermediates 6-iodo-4-chloroquinazolines 2a–d, were heated under reflux temperature with phosphorus oxychloride in the presence of acid acceptor, *N,N*-dimethylaniline. Sulfanilamide or sulfathiazole was then condensed with the corresponding 6-iodo-4-chloroquinazolines 2a–d in ethanol under reflux conditions in the presence of a base catalyst. The IR spectra of compounds 3a–e showed two characteristic absorption bands for the SO_2_ group at 1375 and 1188 cm^−1^ in addition to NH and NH_2_ in the region 3410–2337 cm^−1^ and additional absorption bands of 2 NH groups in the region 3310–3208 cm^−1^ for compounds 3a–e as well as results of C^13^ analysis (Fig. S1, supplementary data). Their corresponding ^1^H NMR revealed the two NH and NH_2_ D_2_O exchangeable signals at 11.0–12.0 ppm, respectively (Fig. S2). The mass spectra revealed the corresponding molecular ion in each case.

### In vitro anti-cancer activity

As displayed in Table 1, four compounds, 3a–d, were found effective against the four tested tumor cell lines. However, compound **3e** was devoid of anticancer activities. The most effective compound was 3c, with sulfanilamide at position 4 and 4-methoxyphenyl at position 2. As shown in Table 2, it inhibited carbonic anhydrase CAXII at the IC_50_ of 3.69 µM, and it positively stopped the growth of all four tested tumor cells at concentrations ranging from 4.0–8.0 µM. The second interesting compound, 3b, was also able to stop the proliferation of the four tumor cell lines at a somewhat lower concentration, the IC_50,_ which was between 6.0–9.0 µM, and it inhibits CAXII activity at a concentration 3.69 µM (Table 2).

**Table 1:** In vitro antitumor activities of the five newly synthesized compounds against the tested cell lines

In vitro antitumor activities (IC_50_ µM)					
Compound	HeLa (Cervical)	MDA MB231 (Breast)	MIA PaCa2 (Pancreatic)	NCI H1975 (non-small cell Lung)	A4 Cell line (Control cell line)
3a	10.5 ± 1.6	12.0 ± 1.25	14 ± 1.60	13.2 ± 1.50	> 50
3b	6.1 ± 0.12	7.3 ± 0.80	7.3 ± 0.90	9.1 ± 1.8	> 50
3c	4.0 ± 0.66	6.4 ± 0.90	5.5 ± 0.80	8.0 ± 0.50	> 50
3d	8.2 ± 2.10	6.4 ± 1.76	6.1 ± 1.30	12.3 ± 0.9	> 50
3e	> 50	> 50	> 50	> 50	> 50
Doxorubicin (positive control)	3.25 ± 0.5	2.7 ± 0.6	2.3 ± 0.6	2.8 ± 0.5	ND

*ND* Not determined

**Table 2:** Carbonic anhydrase (CAXII) inhibitory activity

Tested compounds						
CAXII inhibitory activity (IC_50_ µM)	Acetazolamide	3a	3b	3c	3d	3e
	16.25	9.51	3.75	3.69	8.19	> 50

### Antibacterial and antifungal activities

The results of the antibacterial and antifungal activities of the newly synthesized compounds in terms of average inhibition zone (mm) and MIC (µg/mL) are displayed in Table 3. Results showed that the tested compounds have mostly a broad spectrum of activity as well as antifungal activity. However, they have better antibacterial activity against Gram-positive *S. aureus* as compared to Gram-negative strains (Table 3). Compound 3c exerted the highest antibacterial and antifungal activity as compared to the other tested compounds. As shown in Table 3, the lowest antibacterial activities of all the tested compounds were observed against *P. aeruginosa*.

**Table 3:** Antimicrobial activities of the five newly synthesized compounds against the tested standard microbial strains

Compound	Inhibition Zone (mm)					MIC (µg/mL)				
	SA ATCC 25913	EC ATCC 25922	PA ATCC 27853	KP ATCC 49619	CA ATCC 14053	SA ATCC 25913	EC ATCC 25922	PA ATCC 27853	KP ATCC 49619	CA ATCC 14053
3a	14	10	0	10	10	8.0	8.0	ND	16	16
3b	18	12	10	12	10	2.0	8.0	32	32	16
3c	22	15	12	14	16	1.0	4.0	16	4.0	4.0
3d	16	10	12	12	12	4.0	32	32	8.0	8.0
3e	14	10	10	10	12	16	32	32	32	16
Ciprofloxacin	21	25	25	24	ND	0.5	0.25	0.25	0.25	ND
Fluconazole	ND	ND	ND	ND	20	ND	ND	ND	ND	1.0

SA, *Staphylococcus aureus*; EC, *Escherichia coli*; PA, *Pseudomonas aeruginosa*; KP, *Klebsiella pneumoniae*; CA, *Candida albicans*; MIC, minimum inhibitory concentration; ND, not determined

### Computational analysis and molecular docking

#### Computational analyses

##### ADMET prediction

**Fig. 3:**
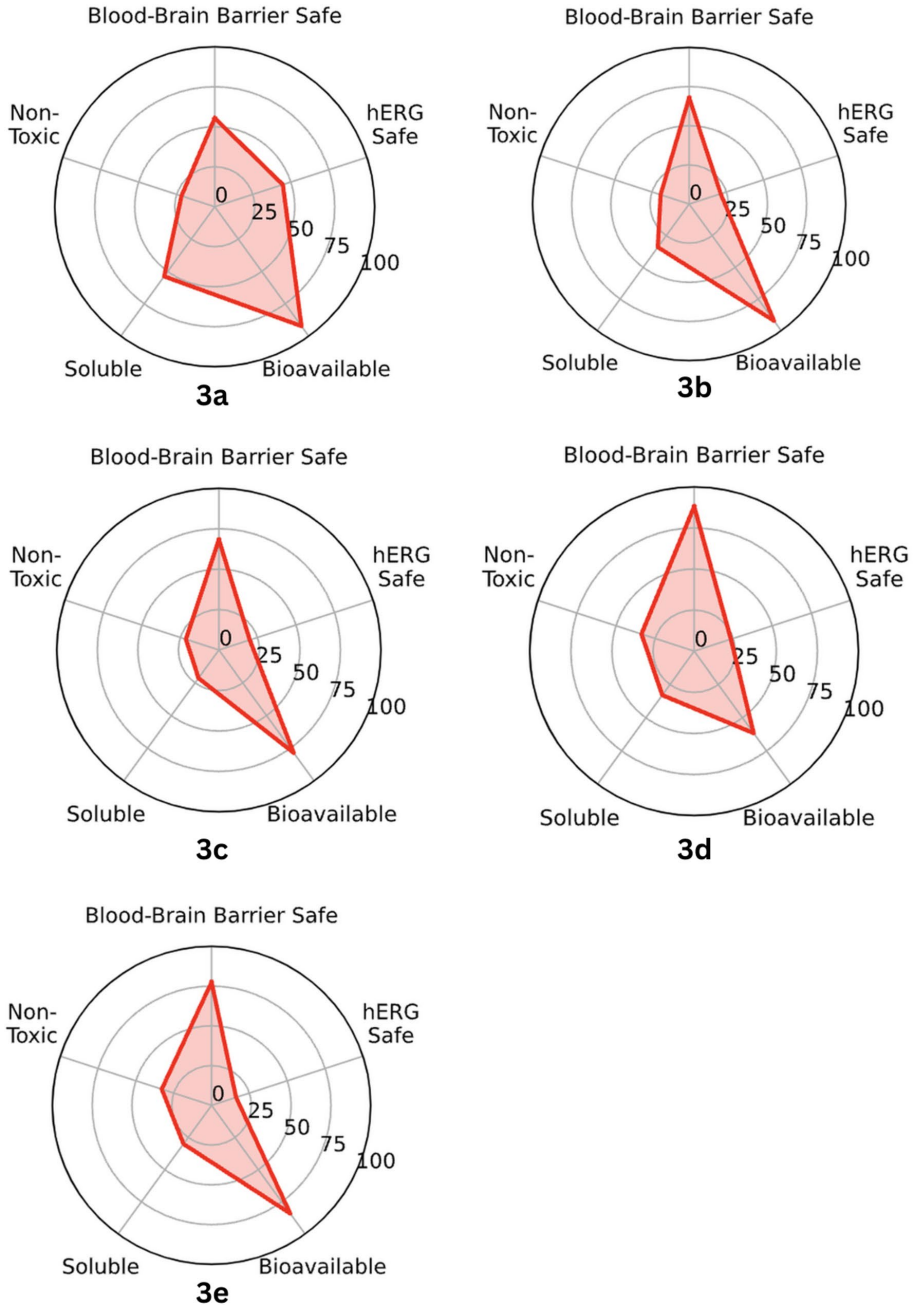
Summary of Absorption, distribution, metabolism, excretion, and toxicity (ADMET) prediction for compound 3a–e

The predicted physicochemical properties of compound 3a–e are displayed in Table 4. A summary of absorption, distribution, metabolism, excretion, and toxicity (ADMET) prediction is displayed in Fig. 3. Results showed that compound 3d has the highest molecular weight (601.45 g/mol). Regarding lipophilicity, the results showed that the five compounds were arranged in descending order as follows: 3e, 3c, 3d, 3b, and 3a. All compounds have 2 hydrogen bonds donors except 3d, which has a 3. For the topological polar surface area (TPSA), compound 3d has the highest TPSA, followed by 3e, 3b, 3c, and 3a in descending order. The in silico pharmacokinetic evaluation of compounds 3a–e, based on five key parameters—Blood–Brain Barrier (BBB) Safety, hERG Safety (Cardiac Toxicity), General Toxicity, Solubility, and Bioavailability—revealed some distinct drug-likeness properties for each derivative (Fig. 3). Compounds 3c, 3d, and 3e exhibited high BBB safety, suggesting their potential for CNS penetration, whereas compounds 3a and 3b displayed moderate BBB permeability, indicating a reduced likelihood of CNS-related effects. All five compounds showed moderate hERG safety, suggesting a manageable risk of cardiac toxicity that would require further validation. In terms of toxicity, compounds 3c, 3d, and 3e demonstrated lower toxicity risk, making them strong candidates for further drug development, while compounds 3a and 3b exhibited moderate toxicity. Despite this, all compounds exhibited moderate bioavailability, suggesting they can achieve systemic circulation but may require optimization for enhanced drug efficacy. Overall, compound 3e emerged as the most promising candidate, with high BBB safety, low toxicity, and moderate bioavailability, followed by compounds 3c and 3d, which also displayed strong potential. However, the poor solubility of all derivatives remains a critical challenge that must be addressed through formulation modifications to optimize their therapeutic viability.

**Table 4:** Physicochemical properties of the compound 3a–e

Compounds	3a	3b	3c	3d	3e
Molecular Weight (g/mol)	426.24	532.36	595.45	601.45	645.50
Lipophilicity (LogP)	2.63	4.30	5.90	5.61	5.92
Hydrogen Bond Acceptor	5	6	7	8	9
Hydrogen Bond Donor	2	2	2	3	2
Linpiski rule of 5	4	3	2	2	2
Quantitative Estimate of Drug likeness (QED)	0.63	0.37	0.24	0.22	0.20
Topological Polar Surface Area (TPSA)	97.97	107.20	96.87	117.10	115.33

### Binding affinities towards target receptors

**Fig. 4:**
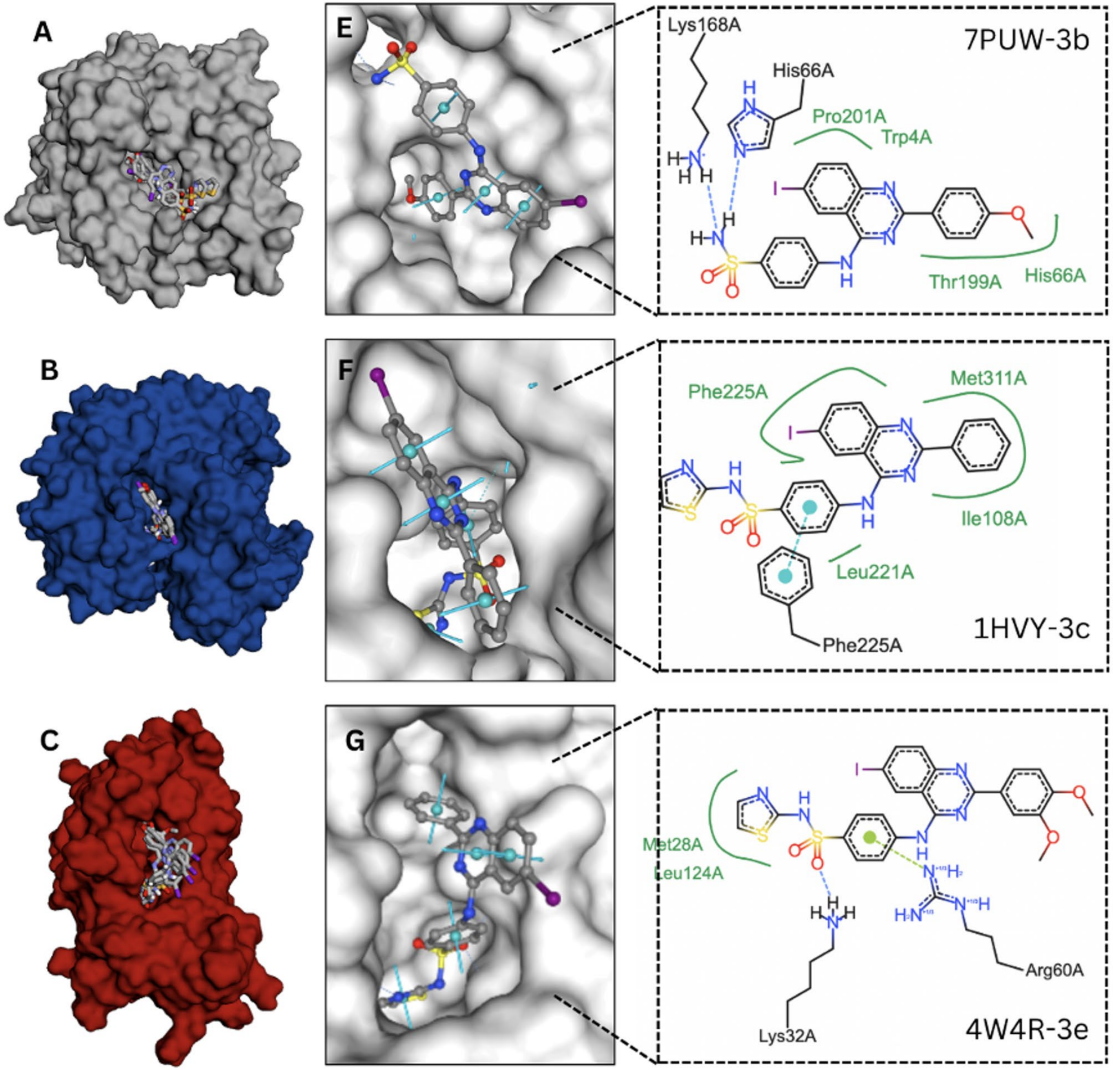
Docking analysis of 3a–e compounds to the active sites of carbonic anhydrase XII, hCAXII (**A**), thymidylate synthase, hTS (**B**), thymidylate kinase, hTK (**C**). The top compound, 3c, 3b and 3c, is shown individually in the active site (**E**, **F**, **G**), and presented in 2D structural interactions with the binding site residues for each target protein, respectively

**Fig. 5:**
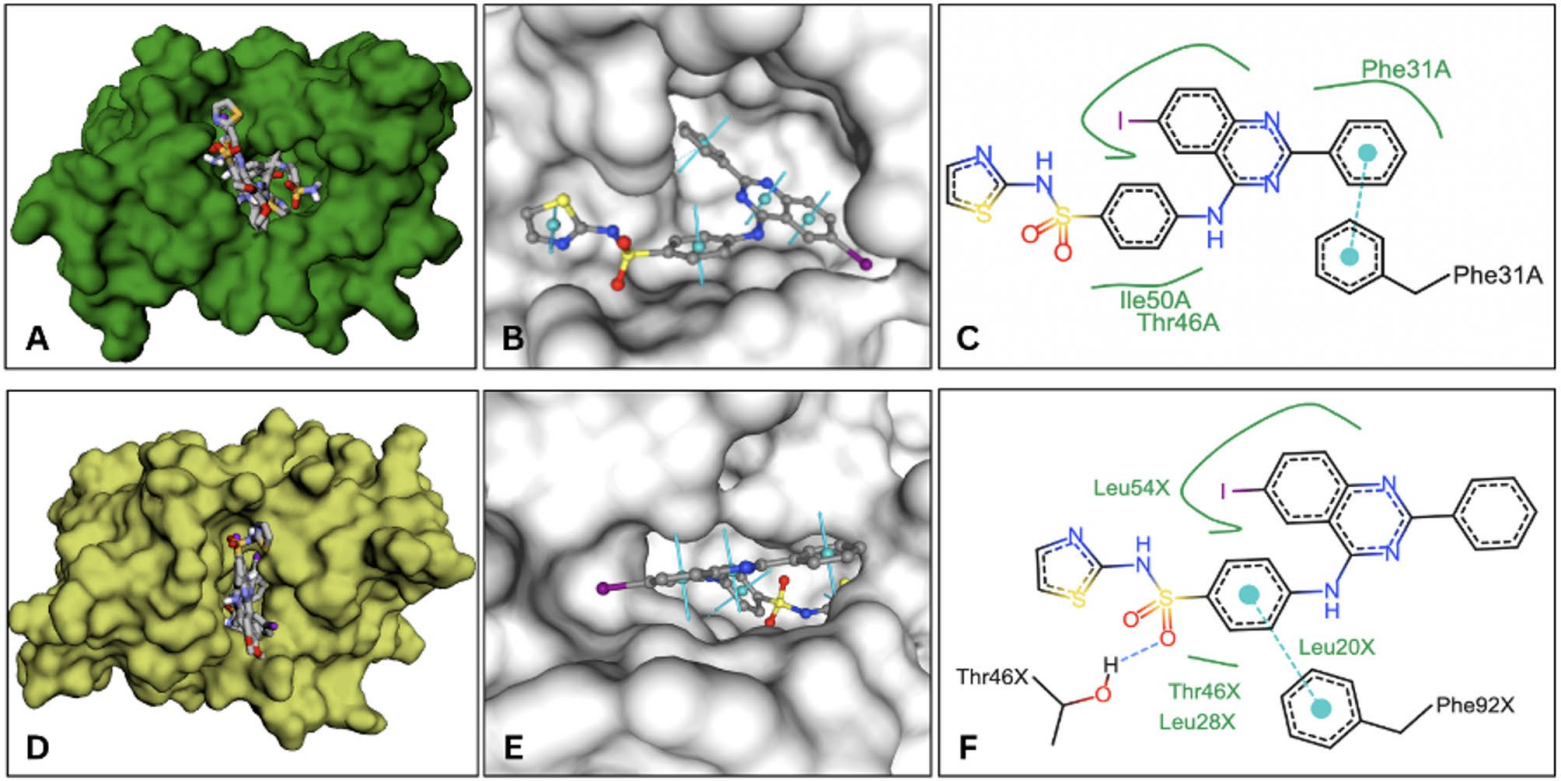
Docking analysis of 3a–e compounds to the active sites of dihydrofolate reductase of (**A**) *E. coli* and (**D**) *S. aureus.* The top compound, 3c, is shown individually in the active site (**B**, **E**), and presented in 2D structural interactions with the binding site residues (**C**, **F**), respectively

Molecular docking studies were conducted to assess the binding affinities of compounds 3a–e against four human target proteins and one bacterial target protein. The docking results, summarized in Table 5, reveal the interaction strengths of these compounds with human CAXII, hTS, hTK, and DHFR of *E. coli* and *S. aureus*. The 3D visualization of the docked compounds to the target receptors is shown in Figs. 4 and 5.

**Table 5:** Docking binding affinities of 3a–e compounds to the target receptors. Additional docking analyses of known inhibitors to the target receptors were performed for reference purposes

Compounds	Binding Affinities (kcal/mol)				
	Human carbonic anhydrase XII, CAXII (PDB 7PUW)	Human thymidylate synthase, hTS (PDB 1HVY)	Human thymidine kinase, hTK (PDB 1W4R)	*E. coli* dihydrofolate reductase, DHFR (PDB 1DDR)	*S. aureus* dihydrofolate reductase, DHFR (PDB 3FRD)
Redocking	− 5.92	− 6.58	− 10.84	− 6.37	− 4.11
3a	− 7.12	− 8.10	− 9.22	− 8.55	− 8.86
3b	− 8.45	− 9.59	− 9.02	− 8.87	− 9.63
3c	− 8.33	− 9.96	− 9.15	− 10.52	− 10.24
3d	− 8.03	− 9.90	− 9.26	− 10.04	− 9.81
3e	− 8.01	− 9.40	− 10.16	− 9.14	− 9.7
Acetazolamide	− 5.98	NA	NA	NA	NA
Methazolamide	− 6.43	NA	NA	NA	NA
Dorzolamide	− 6.95	NA	NA	NA	NA
Fluorouracil	NA	− 4.89	NA	NA	NA
Pemetrexed	NA	− 8.88	NA	NA	NA
Nolatrexed	NA	− 7.63	NA	NA	NA
Stavudine	NA	NA	− 7.98	NA	NA
Aciclovir	NA	NA	− 7.35	NA	NA
Trimethoprim	NA	NA	NA	− 7.18	− 7.43

PDB, Protein Data Bank; NA, not applicable

Docking studies against CAXII revealed an average binding affinity of − 8.00 ± 0.52 kcal/mol across the five compounds. Among these, compound 3b demonstrated the strongest interaction, achieving a docking score of − 8.45 kcal/mol, while compound 3a exhibited the weakest binding affinity at − 7.12 kcal/mol. To further contextualize these findings, additional docking simulations were performed with known carbonic anhydrase inhibitors, namely acetazolamide, methazolamide, and dorzolamide. These CAXII inhibitors displayed lower binding affinities (− 6.45 ± 0.49 kcal/mol) compared to compounds 3a–e, highlighting the binding potential of the novel compounds. Intermolecular analysis of compound 3b with hCAXII revealed key hydrogen bond interactions involving Lys168 and His66 with the sulfonamide group, which are critical for anchoring the compound in the active site (Fig. 4A). Meanwhile, the other functional groups contributed to stabilizing the compound within the binding pocket through hydrophobic interactions with residues such as Pro202, Trp4, Thr199, and His66 (Fig. 4E). The binding of compound 3b is consistent with the predominantly hydrophobic nature of CAXII catalytic pocket, which not only enhances the binding affinities of hydrophobic compounds but also helps maintain the structural integrity of the receptor-ligand complex. These results highlight the promising potential of compound 3b as a potent CAXII inhibitor.

Compared to hCAXII, binding of compound 3a–e to hTS exhibited higher average binding affinities of − 9.39 ± 0.76 kcal/mol. Notably, compound 3c emerged as the top binder with a score of − 9.96 kcal/mol, closely followed by compound 3d at − 9.90 kcal/mol. Compound 3a, however, displayed the least favorable interaction with a docking score of − 8.10 kcal/mol. For comparison, hTS also docked with its known inhibitors—fluorouracil, pemetrexed, and nolatrexed. The highest binding affinities exhibited was pemetrexed (− 8.88 kcal/mol), followed by nolatrexed (− 7.63 kcal/mol), and the weakest was fluorouracil (− 4.89 kcal/mol). Overall, other than 3a, the other quinazoline derivatives show a higher affinity for hTS than the known inhibitors. The strong affinity of compound 3c to hTS was driven mainly by the hydrophobic attraction from residues inside the binding pocket which are Phe225, Met311, Ile108, and Leu221 (Fig. 5B). In addition, the fused bicyclic system in the quinazoline core participated in π-stacking interactions with Phe225, which may enhance the binding affinity and orientation of the compound within the catalytic pocket (Fig. 5F).

The binding affinities of compounds 3a–e for hTK were comparable to those observed for hTS, with an average of -9.36 ± 0.46 kcal/mol. Among the tested compounds, compound 3e exhibited the strongest interaction, achieving a docking score of − 10.16 kcal/mol, followed by compound 3d with a score of − 9.26 kcal/mol. In contrast, compound 3b displayed the lowest binding affinity at − 9.02 kcal/mol. For hTK, two antiviral compounds that function as substrate analogs were evaluated. Both compounds, stavudine and aciclovir, were bound to the hTS active site with lower binding affinities (− 7.98, − 7.35 kcal/mol, respectively), which indicate compounds 3a–e are potentially higher to bind to hTS. The sulfonamide group of the compound 3e forms a critical hydrogen bond with the side chain of Lys32, a positively charged residue in the catalytic site (Fig. 4C). This polar interaction plays a crucial role in anchoring the compound and stabilizing its positioning within the active site. Additionally, the guanidinium group of Arg60 formed electrostatic interactions with the nitrogen atoms on the quinazoline moiety of compound 3e (Fig. 4G). These interactions further enhanced the binding affinity by aligning the compound within the catalytic pocket. Hydrophobic attractions between residues Met28 and Leu124 and the sulfonamide-benzene ring moiety of compound 3e contributed additional stability to the ligand-receptor complex. These hydrophobic interactions complement the polar and electrostatic forces, ensuring robust binding and optimal stabilization of the compound within the catalytic site of hTK.

The binding affinity evaluation of compounds 3a–e was conducted against dihydrofolate reductase (DHFR) from *E. coli* and *S. aureus*. Similar to the results observed with the human target proteins, docking studies revealed strong binding affinities of all the compounds to the active site of DHFR. Furthermore, the binding affinity values followed a consistent trend between *E. coli* and *S. aureus*, with compound 3c is the top binder (− 10.52 kcal/mol and − 10.24 kcal/mol, respectively), followed by 3e, 3d, 3b, and 3a exhibiting the lowest binding affinity (− 8.55 kcal/mol and − 8.86 kcal/mol, respectively). For comparison, additional docking analysis was performed using trimethoprim, a well-known DHFR inhibitor, which exhibited significantly lower binding affinities (− 7.18 kcal/mol and − 7.43 kcal/mol). This benchmark further highlights the potential of compounds 3a–e as promising antimicrobial agents, potentially superior to trimethoprim in targeting bacterial DHFR. The binding interactions of these compounds are primarily driven by the hydrophobic environment of the active site (Fig. 5). In *E. coli* DHFR, compound 3c exhibits key hydrophobic interactions with Phe31, Thr46, and Ile50, which contribute to its strong binding. Similarly, in *S. aureus* DHFR, compound 3c interacts with Leu20, Leu28, Thr46, Leu54, and Phe92. Additionally, a hydrogen bond is formed between Thr46 and the sulfonamide group of 3c, which is positioned deep inside the active site, which may enhance the binding stability. This finding highlight compound 3c, a particularly potent candidate for targeting bacterial DHFR. In summary, compound 3c stands out as the most promising compound overall due to its consistently high binding affinities and robust interactions across multiple targets, particularly for hTS, *E. coli* DHFR, and *S. aureus* DHFR.

## Discussion

This study aimed at synthesis and biological evaluation of five novel di-substituted 4-anilinoquinazoline derivatives followed by testing their ability to inhibit CAXII and evaluating their antiproliferative as well as their antimicrobial properties. The respective newly synthesized compounds were 6-Iodo-4-(4-aminosulphonylphenyl-amino) quinazoline (coded 3a), 6-Iodo-4-(4-aminosulphonylphenyl-amino)-2-(4-methoxyphenyl)-quinazoline (coded 3b), 6-Iodo-2-phenyl-4-(4-(thiazol-2-yl) aminosulphonylphenyl-amino) quinazoline (coded 3c), 6-Iodo-2-(4-methoxyphenyl)-4-(4-(thiazol-2-yl) aminosulphonylphenyl-amino)-quinazoline (coded 3d), and 6-Iodo-2-(3,4-dimethoxyphenyl)-4-(4-(thiazol-2-yl) aminosulphonylphenyl-amino)-quinazoline (coded 3e). The principal intermediates 6-iodo-4-chloroquinazolines 2a–d, were heated under reflux temperature with phosphorus oxychloride in the presence of acid acceptor, *N,N*-dimethylaniline (Alafeefy et al. 2016; Ferraroni 2024; Holly et al. 2024). The corresponding 6-iodo-4-chloroquinazolines 2a–d were then condensed with the sulfanilamide or sulfathiazole in ethanol under reflux conditions with sodium carbonate present. In addition to NH and NH_2_ in the area, 3410–2337 cm^−1^ and additional absorption bands of two NH groups in the region 3310–3208 cm^−1^, the infrared spectra of compounds 3a–e revealed two distinctive absorption bands for the SO_2_ group at 1375 and 1188 cm^−1^. The two NH and NH_2_ D_2_O exchangeable signals were detected at 11.0 and 12.0 ppm, respectively, by their matching 1H NMR. In each instance, the mass spectra showed the matching chemical ion.

Additionally, compound 3c reacted with phenyl isothiocyanate in refluxing dry acetone with dry potassium carbonate to form the sulfonyl thioureas derivatives. 4. The absorption of thiourea at 1720 cm^−1^ and the C = S group caused these compounds' infrared spectra to display two bands at 1380 cm^−1^ and 1190 cm^−1^. The 1HNMR of these compounds revealed three D_2_O exchangeable signals of three NH protons at 8.80, 9.70, and 11.80. Each of the final compounds' mass spectra showed a peak that corresponded to the molecular ion. Furthermore, it was unearthed that four compounds, 3a–d, were efficient against the four tumor cell lines that were investigated. Based on the docking results, compounds 3a–e are more potent to the human target receptors compared to known cancer inhibitors. Compounds 3b-e have similar affinity strength to CAXII and hTK, while 3a has the weakest affinity. However, for the hTK compound 3e has the strongest affinity; in contrast, 3b is the weakest. Through hydrophobic interactions with residues like Pro202, Trp4, Thr199, and His66, the additional functional groups helped to stabilize compound 3b within the binding pocket. Compound 3b's binding is in line with CAXII's catalytic pocket's predominately hydrophobic characteristics, which improve hydrophobic compounds' binding affinities while also preserving the receptor- ligand complex's structural integrity. These findings demonstrate compound 3b's encouraging potential as a strong CAXII inhibitor. On the other hand, compound 3e binding affinity is the highest for thymidine kinase, however, this activity was not reflected in vitro. Therefore, this unaligned finding should be reinvestigated. Compound 3c, which has 4-methoxyphenyl at position 2 and sulfanilamide at position 4, was the most efficacious chemical. Our findings demonstrated that at doses ranging from 4.0 to 8.0 µM, compound 3c favorably suppressed the development of all four tested tumor cells and inhibitedCAXII at an IC50 of 3.69 µM. However, at a little lower dosage, the second intriguing chemical, 3b, was similarly able to block the growth of the four tumor cell lines. Its IC50 was between 6.0 and 9.0 µM, and at 3.69 µM, it suppresses CAXII activity, suggesting that it has promising anticancer effects.

Regarding the structure–activity relationship, we noticed that compound 3a (with no substitution at position-2) was weaker than 3b which contained a 4-methoxyphenyl group at position-2, keeping in both cases sulfanilamide at position-4 of the quinazoline backbone. However, modifying compound 3b just by removing 4-methoxy, keeping phenyl at position-2 with thiazolyl as R_1_ improved the activity, as evidenced by compound 3c, which inhibited the tumor at a lower concentration. This slight improvement was reduced in the case of compound 3d, which has 4-methoxyphenyl at position 2 with thiazolyl as R_1_ of the quinazoline nucleus. Although sulfanilamide was kept at position 4, maybe the methoxy group at position 3 hindered the compound from binding with the receptor (Alafeefy et al. 2016; Shagufta and Ahmad 2017; Yu and Hung 2022). The earlier improvement in activity by compound 3c was lost when we used sulfathiazole instead of sulfanilamide, 3e which has 3,4-dimethoxyphenyl at position 2 of the quinazoline nucleus. On the other hand, compound 3c proved to be the most suitable one for further studies to explore the exact mechanism of action. Also, we noticed that the CAXII inhibitor activity lies well in line with the observed antitumor one. Most probably illustrating one of the potential mechanisms of action. 3c inhibited CAXII at 3.69 nM, 3b at 3.75 nM, 3d 8. 91 nM followed by 3a at 9.51 nM (Meşeli et al. 2021). Interestingly, this lab analysis is aligned with the docking results. It showed higher binding affinity (− 8.33 kcal/mol) as compared to the three standard CAXII inhibitors (5.98–6.95 kcal/mol).

The newly synthesized compounds' antibacterial and antifungal properties were tested and analyzed in terms of their average inhibition zone (mm) and minimum inhibitory concentration (MIC) (µg/mL). The investigated compounds exhibited a broad range of activity and antifungal activity, according to the results; nevertheless, they exhibited superior antibacterial activity against Gram-positive *S. aureus* strains in contrast to Gram-negative strains. Remarkably, when compared to the other compounds examined, compound 3c had the most antibacterial and antifungal activity. Interestingly, this lab analysis is aligned with the docking results. It showed a higher binding affinity of − 10.53 and 10.24 kcal/mol as compared to trimethoprim, the standard DHFR inhibitor that showed − 7.18 and − 7.43, kcal/mol, for *E. coli* and *S. aureus*, respectively. According to our findings, *P. aeruginosa* exhibited the lowest antibacterial activity of any chemical investigated. Interestingly, several studies have reported that the antibacterial activities of 2,4-disubstituted quinazoline analogs have been proven to be more active against Gram-positive as compared to Gram-negative bacteria (Megahed et al. 2022). The newly synthesized quinazolinone derivatives possess antimicrobial properties, particularly against the Gram-positive strains and *C. albicans,* because of their ability to interact with the microbial cell wall, DNA structures, or histidine kinases as previously reported (Kumar Pandey et al. 2021; Sulthana et al. 2021; Nasr et al. 2022; Ishikawa et al. 2024).

Interestingly, the antimicrobial activities of quinazolinone derivatives are enhanced by substitutions 2 and 3, the presence of halogen atoms at 6 and 8 positions, and substitution (primarily amine or substituted amine) at the 4th position of the quinazolinone ring (Ghorab et al. 2013; Zayed and Hassan 2014; Maestri et al. 2017). These findings have been reported in various literature studies on the structure–activity relationship of quinazolinone derivatives (Devi et al. 2012; El-Sayed et al. 2021). Antimicrobial properties need the presence of methyl, amine, or thiol groups at position 2 and a substituted aromatic ring at position 3 (Maestri et al. 2017). Our results are aligned with Zayed et al., who synthesized several quinazolinone compounds substituted with N3-sulfonamide. They found that the antibacterial activity of quinazolinone was greatly increased when iodine was substituted for the primary aromatic ring at positions 6 and 8 (Zayed and Hassan 2014). Kumar and colleagues carried out he synthesis and assessment of new antimicrobials, 2-(chloromethyl)-3-[(E)-phenyldiazenyl]-4-methyl-6-oxo-5- the derivatives of -2-thioxo-5,6-dihydropyrimidine-1(2H)-yl) quinazoline-4(3H)-ones. They found that the antibacterial analyses showed that the methyl group on the phenyl ring had a major impact on the antibacterial profile when combined with methoxy; additionally, compounds with methyl groups were more active when compared to those containing other groups that donate or withdraw electrons. The findings also showed that this newly synthesized quinazoline-4(3H) was more effective against Gram-positive bacteria (Kumar et al. 2011). Our findings showed that compound c was the best-performing inhibitor, as it had a docking score of − 10.52 kcal/mol, while compound 3d, which had a score of − 10.04 kcal/mol, was not far behind. Thr46, Ile50, and Phe31 hydrophobic contacts were the main drivers of compound 3c's interactions, with π-stacking interactions with Phe31 serving as a supplement. Accordingly, compound 3c is the most promising of all because of its strong interactions and consistently high binding affinities across a variety of targets, especially for hTS, *E. coli* DHFR, and *S. aureus* DHFR. In conclusion, synthesis, structural elucidation, antiproliferative activity, and CAXII inhibitory activity of five new 4-anilino-6-iodo-2-substituted-quinazoline compounds were undertaken. Four compounds 3a–d were effective against the four tested tumor cell lines, and compound 3e showed weak activity. The most effective compounds against the four tested tumor cell lines and for inhibiting CAXII activity were 3c with sulfanilamide at position-4 and 4-methoxyphenyl at position-2 and compound 3b, respectively. The CAXII inhibitory activity lies well with the observed antitumor activity, and this most probably illustrates one of the potential mechanisms of action. The lab results are aligned with docking analysis of the respective compounds against the potential targets including, CAXII, hTS, hTK, for the anticancer activities and DHFR of *E. coli* and *S. aureus* for the antibacterial properties. The respective compounds have a broad spectrum of activity as well as antifungal activity. However, they have better antibacterial activity against Gram-positive as compared to Gram-negative strains. Because of its consistently high binding affinities and strong interactions across a variety of targets, especially for hTS, *E. coli* DHFR, and* S. aureus* DHFR, compound 3c stands out as the most promising chemical overall. It is strongly advised to clinically evaluate compounds 3c and 3b for preclinical and clinical testing as anticancer drugs for possible human application.

## Electronic supplementary material

Below is the link to the electronic supplementary material.

Supplementary Material 1

## Data Availability

All data generated or analyzed during this study are included in this published article and supplementary file.
