# Supplementary material for: Anticancer, antimicrobial and molecular docking analysis of newly synthesized iodoquinazoline derivatives
Source: AMB Express. 2025 Jun 18;15:95. doi: 10.1186/s13568-025-01899-1 (PMC12177125; doi:10.1186/s13568-025-01899-1)
Supplement: Supplementary file 1 — Supplementary Material 1 [file 13568_2025_1899_MOESM1_ESM.pdf]

3a

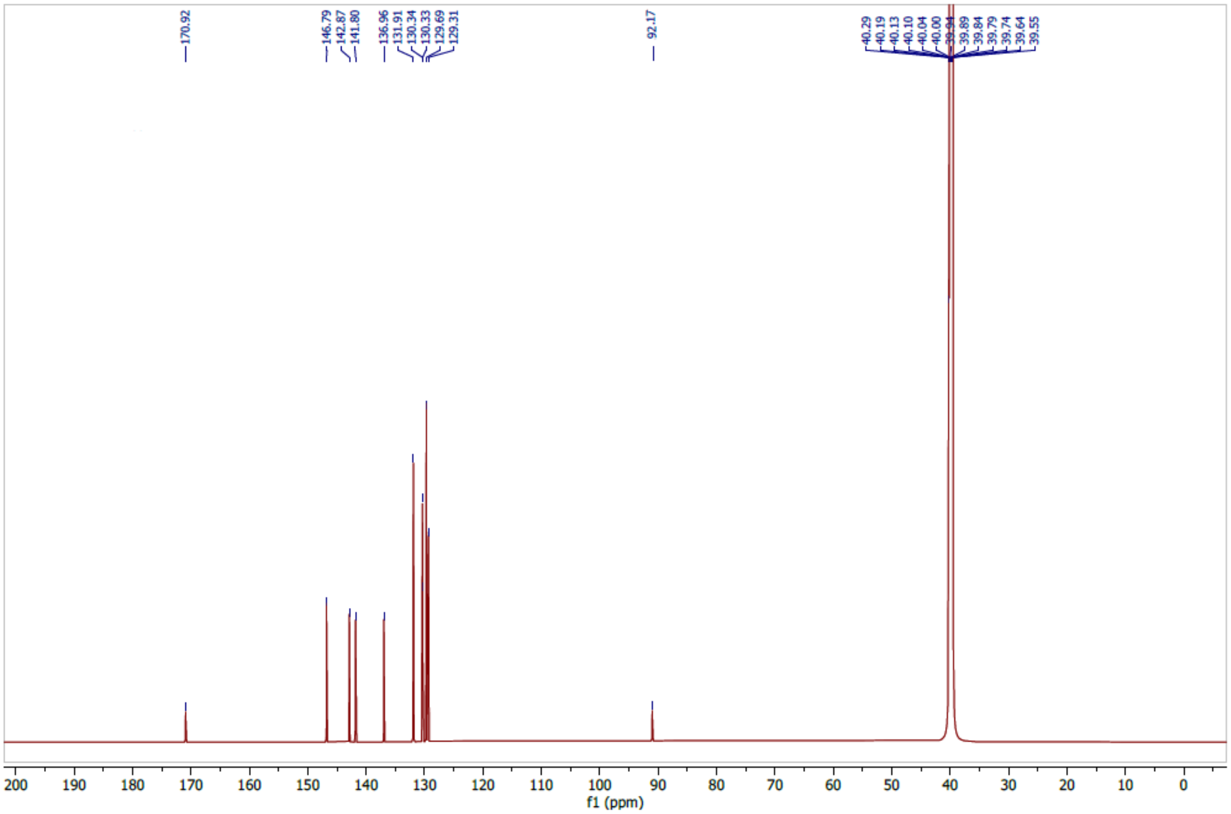

170.92
146.79
142.87
141.80
136.96
131.91
130.34
130.33
129.69
129.31
92.17
40.29
40.19
40.13
40.10
40.04
40.00
39.94
39.89
39.84
39.79
39.74
39.64
39.55
200 190 180 170 160 150 140 130 120 110 100 90 80 70 60 50 40 30 20 10 0
f1 (ppm)

3b

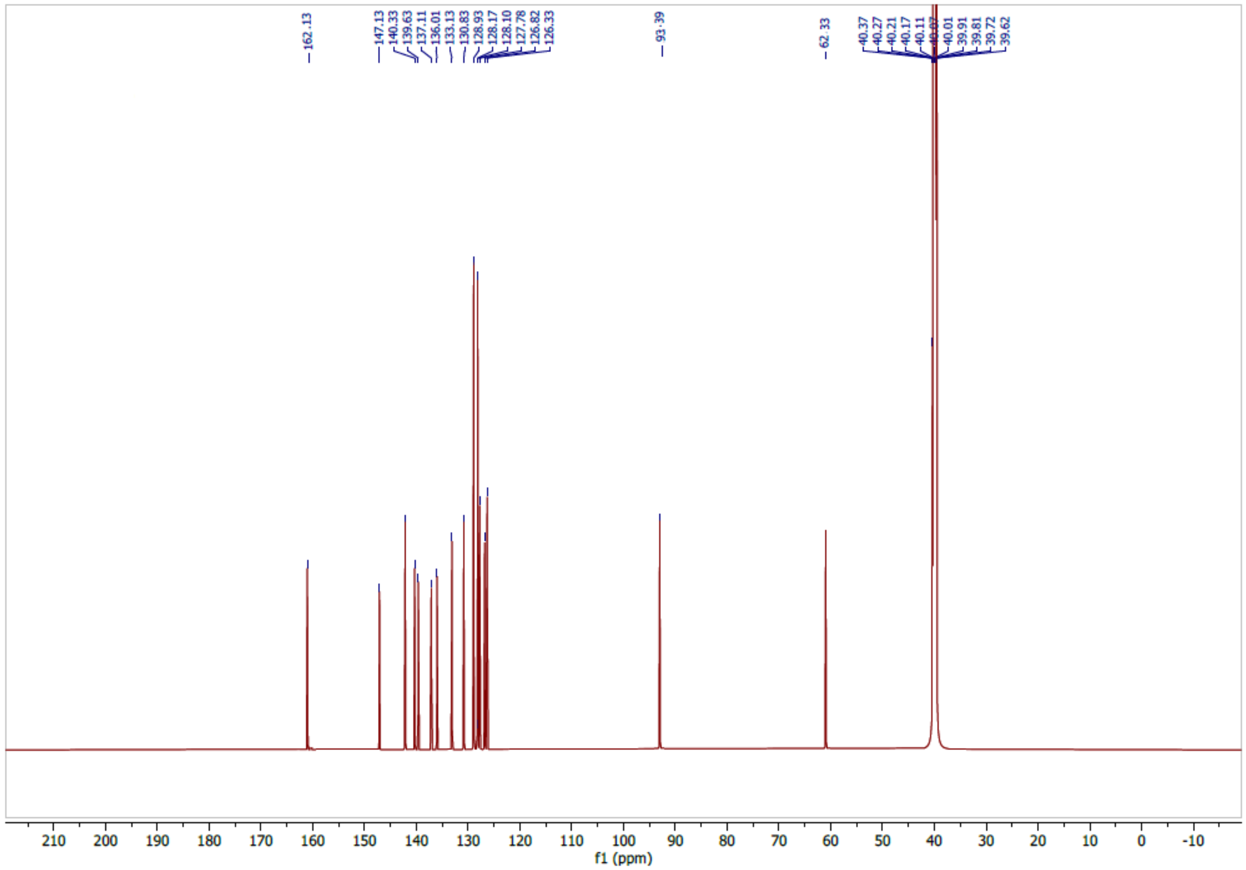

162.13
147.13
140.33
139.63
137.11
136.01
133.13
130.83
128.93
128.17
128.10
127.78
126.82
126.33
93.39
62.33
40.37
40.27
40.21
40.17
40.11
40.07
40.01
39.91
39.81
39.72
39.62
f1 (ppm)

3c

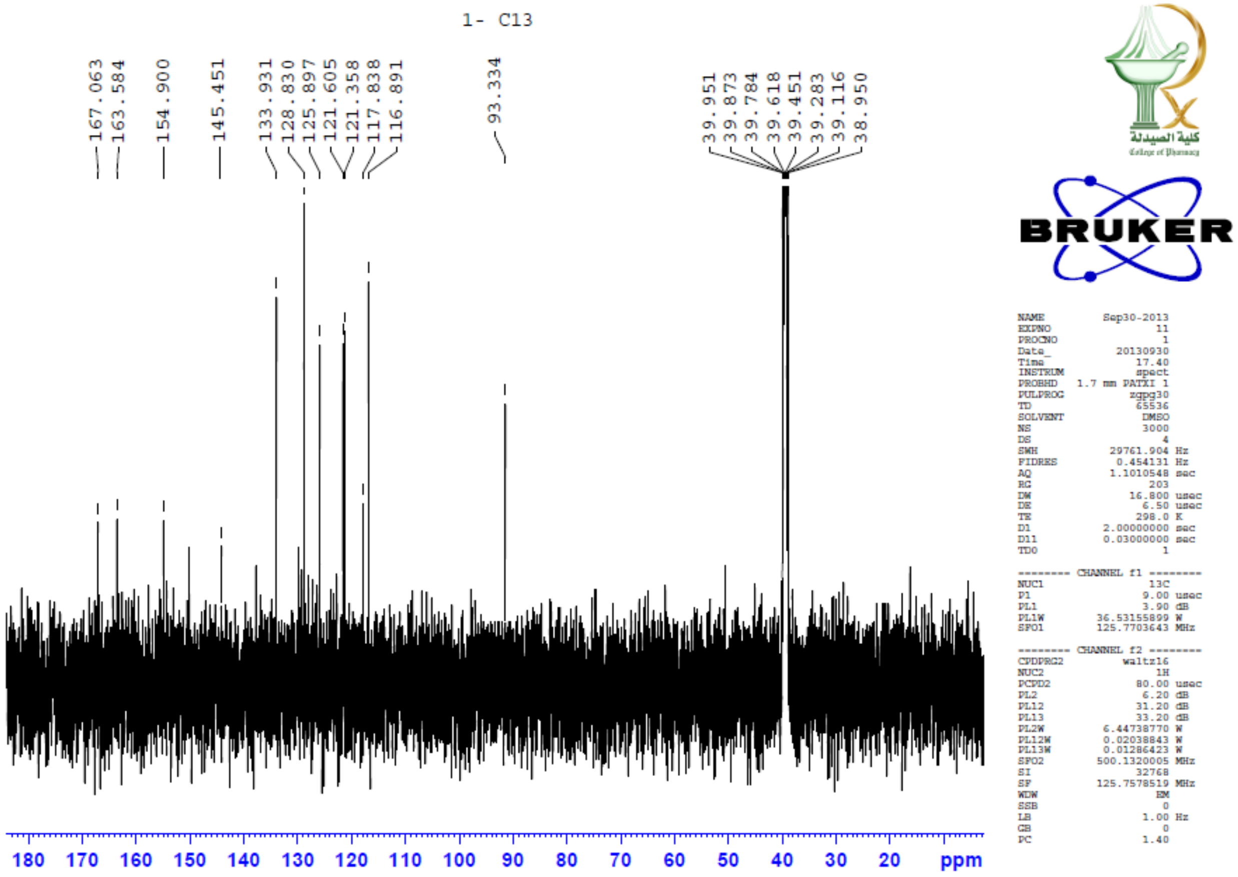
1- C13
167.063
163.584
154.900
145.451
133.931
128.830
125.897
121.605
121.358
117.838
116.891
93.334
39.951
39.873
39.784
39.618
39.451
39.283
39.116
38.950
180 170 160 150 140 130 120 110 100 90 80 70 60 50 40 30 20 ppm
BRUKER
NAME Sep30-2013
EXPNO 11
PROCNO 1
Date_ 20130930
Time 17.40
INSTRUM spect
PROBHD 1.7 mm PATXI 1
PULPROG zgpg30
TD 65536
SOLVENT DMSO
NS 3000
DS 4
SWH 29761.904 Hz
FIDRES 0.454131 Hz
AQ 1.1010548 sec
RG 203
DW 16.800 usec
DE 6.50 usec
TE 298.0 K
D1 2.00000000 sec
D11 0.03000000 sec
TD0 1
======== CHANNEL f1 ========
NUC1 13C
P1 9.00 usec
PL1 3.90 dB
PL1W 36.53155899 W
SFO1 125.7703643 MHz
======== CHANNEL f2 ========
CPDPRG2 waltz16
NUC2 1H
PCPD2 80.00 usec
PL2 6.20 dB
PL12 31.20 dB
PL13 33.20 dB
PL2W 6.44738770 W
PL12W 0.02038843 W
PL13W 0.01286423 W
SFO2 500.1320005 MHz
SI 32768
SF 125.7578519 MHz
WDW EM
SSB 0
LB 1.00 Hz
GB 0
PC 1.40

3d

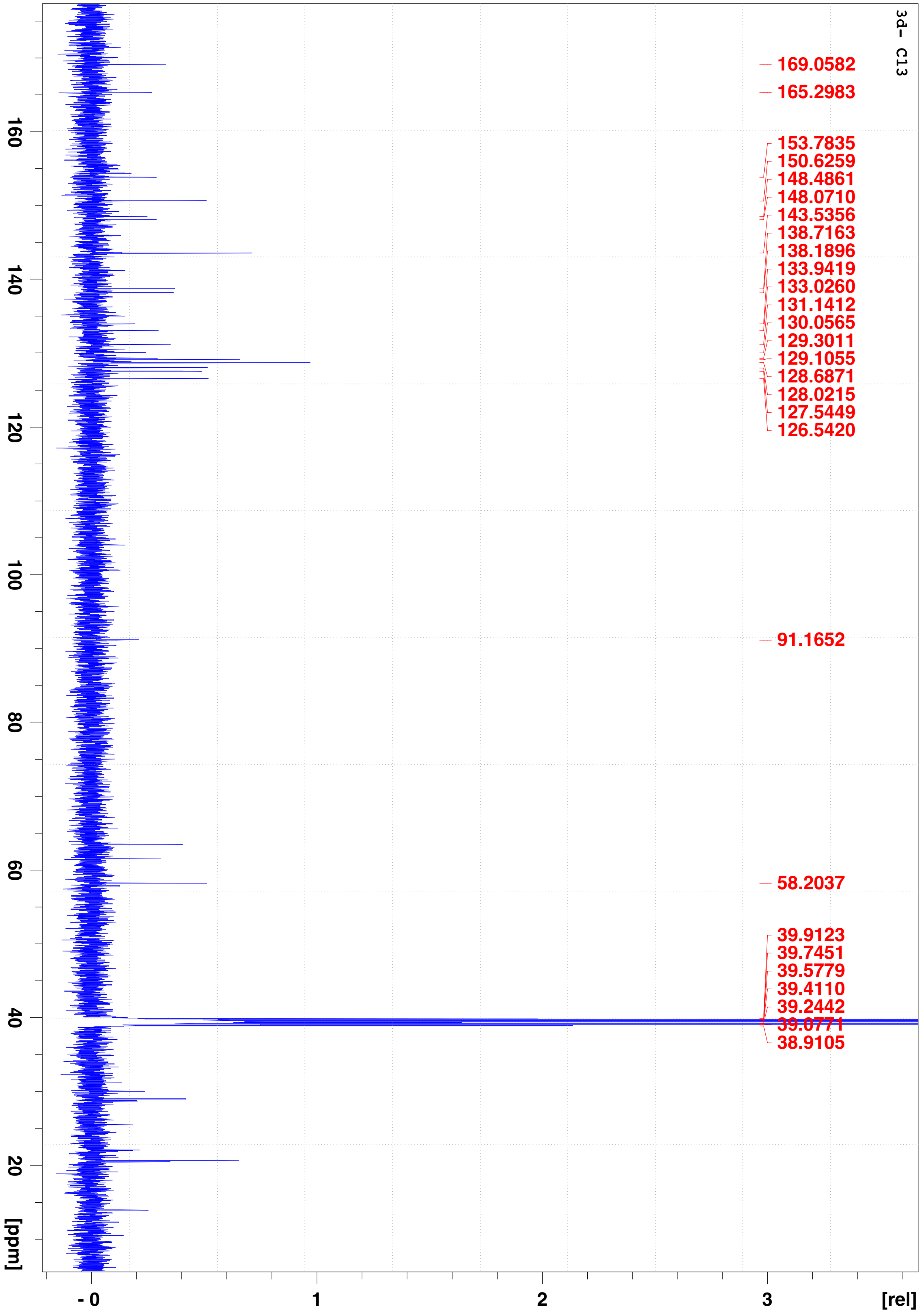
3d– C13
169.0582
165.2983
153.7835
150.6259
148.4861
148.0710
143.5356
138.7163
138.1896
133.9419
133.0260
131.1412
130.0565
129.3011
129.1055
128.6871
128.0215
127.5449
126.5420
91.1652
58.2037
39.9123
39.7451
39.5779
39.4110
39.2442
39.0771
38.9105
160
140
120
100
80
60
40
20
[ppm]
- 0
1
2
3
[rel]

3e

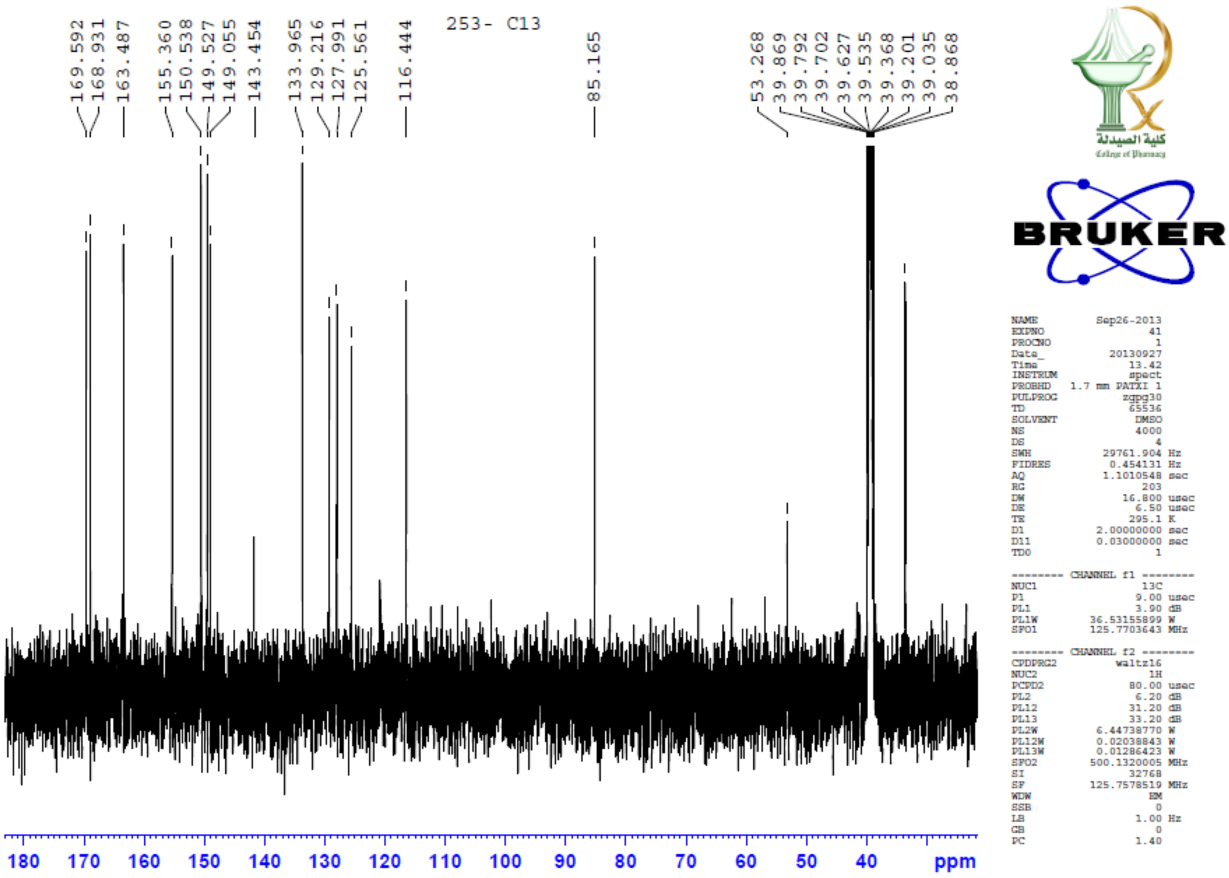

Fig. S1. C13 analysis of the compounds: 3a (6-Iodo-4-(4-aminosulphonylphenyl-amino) quinazoline); 3b (6-Iodo-4-(4-aminosulphonylphenyl-amino)-2-(4-methoxyphenyl)-quinazoline); 3c (6-Iodo-2-phenyl-4-(4-(thiazol-2-yl) aminosulphonylphenyl-amino) quinazoline); 3d (6-Iodo-2-(4-methoxyphenyl)-4-(4-(thiazol-2-yl) aminosulphonylphenyl-amino)-quinazoline); and 3e (6-Iodo-2-(3,4-dimethoxyphenyl)-4-(4-(thiazol-2-yl) aminosulphonylphenyl-amino)-quinazoline).

3a

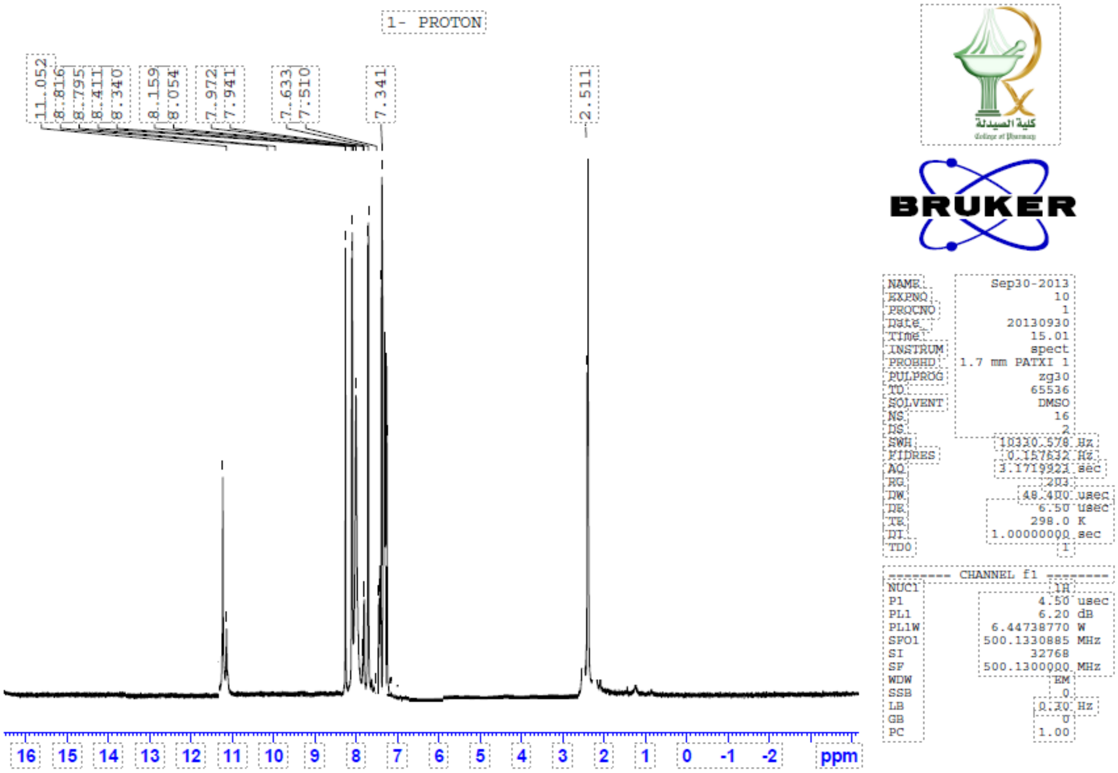
1- PROTON
11.052
8.816
8.795
8.411
8.340
8.159
8.054
7.972
7.941
7.633
7.510
7.341
2.511
16 15 14 13 12 11 10 9 8 7 6 5 4 3 2 1 0 -1 -2 ppm
BRUKER
NAME Sep30-2013
EXPNO 10
PROCNO 1
Date_ 20130930
Time 15.01
INSTRUM spect
PROBHD 1.7 mm PATXI 1
PULPROG zg30
TD 65536
SOLVENT DMSO
NS 16
DS 2
SWH 10330.578 Hz
FIDRES 0.157632 Hz
AQ 3.1719923 sec
RG 203
DW 48.400 usec
DE 6.50 usec
TE 298.0 K
D1 1.00000000 sec
TD0 1
======== CHANNEL f1 ========
NUC1 1H
P1 4.50 usec
PL1 6.20 dB
PL1W 6.44738770 W
SFO1 500.1330885 MHz
SI 32768
SF 500.1300000 MHz
WDW EM
SSB 0
LB 0.30 Hz
GB 0
PC 1.00

3b

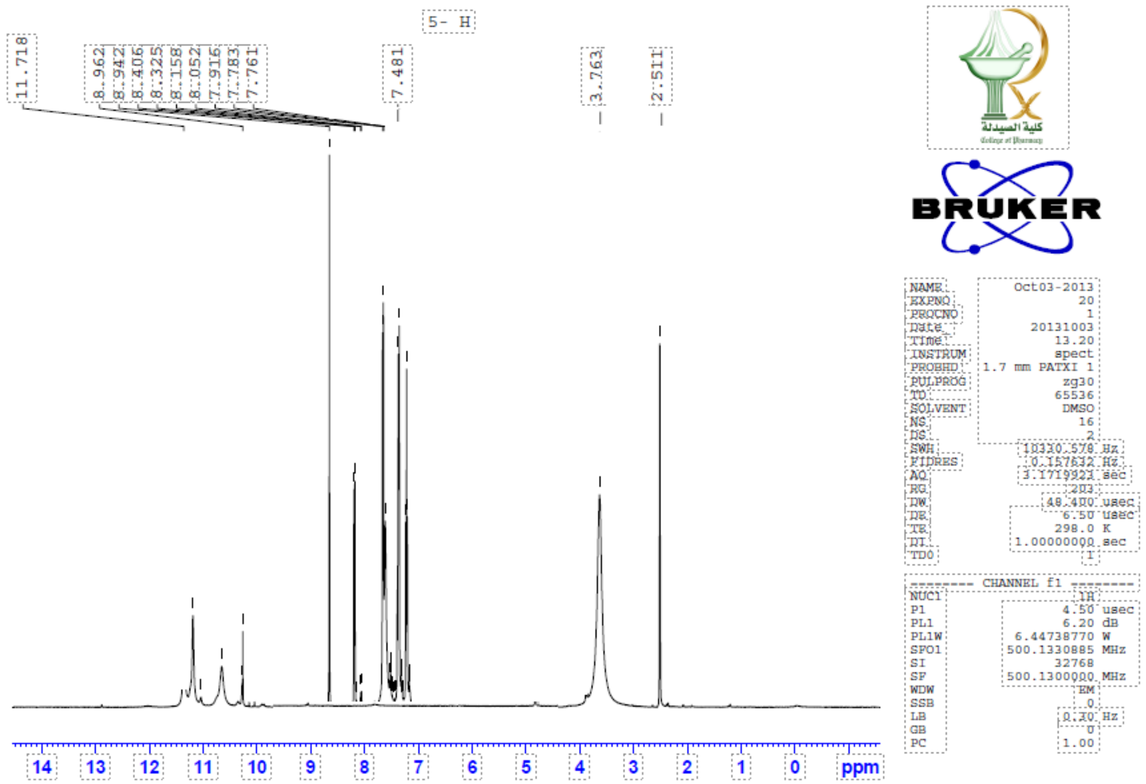
11.718
8.962
8.942
8.406
8.325
8.158
8.052
7.916
7.783
7.761
7.481
5- H
3.763
2.511
14
13
12
11
10
9
8
7
6
5
4
3
2
1
0
ppm
BRUKER
NAME Oct03-2013
EXPNO 20
PROCNO 1
Date_ 20131003
Time 13.20
INSTRUM spect
PROBHD 1.7 mm PATXI 1
PULPROG zg30
TD 65536
SOLVENT DMSO
NS 16
DS 2
SWH 10330.578 Hz
FIDRES 0.157632 Hz
AQ 3.1719923 sec
RG 203
DW 48.400 usec
DE 6.50 usec
TE 298.0 K
D1 1.00000000 sec
TD0 1
======== CHANNEL f1 ========
NUC1 1H
P1 4.50 usec
PL1 6.20 dB
PL1W 6.44738770 W
SFO1 500.1330885 MHz
SI 32768
SF 500.1300000 MHz
WDW EM
SSB 0
LB 0.30 Hz
GB 0
PC 1.00

3c

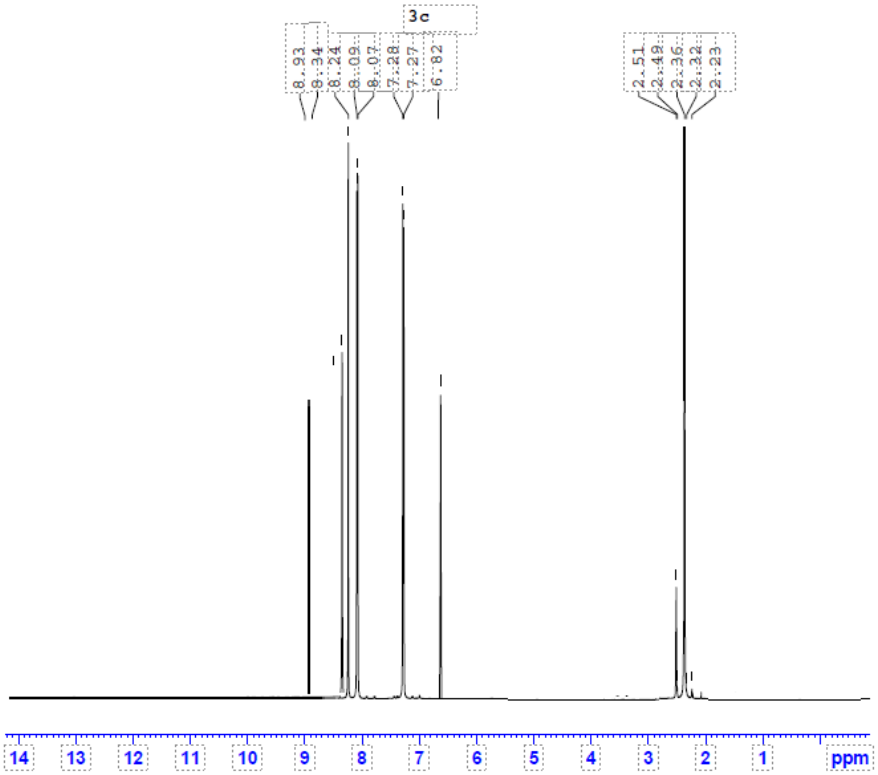

NAME	Oct03-2013
EXPNO	30
PROCNO	1
Date_	20131003
Time	16.03
INSTRUM	spect
PROBHD	1.7 mm PATXI 1
PULPROG	zg30
TD	65536
SOLVENT	DMSO
NS	16
DS	2
SWH	10330.578 Hz
FIDRES	0.157632 Hz
AQ	3.1719923 sec
RG	161
DW	48.400 usec
DE	6.50 usec
TE	298.0 K
D1	1.00000000 sec
TD0	1
======== CHANNEL f1 ========	
NUC1	1H
P1	4.50 usec
PL1	6.20 dB
PL1W	6.44738770 W
SFO1	500.1330885 MHz
SI	32768
SF	500.1300000 MHz
WDW	EM
SSB	0
LB	0.30 Hz
GB	0
PC	1.00

3d

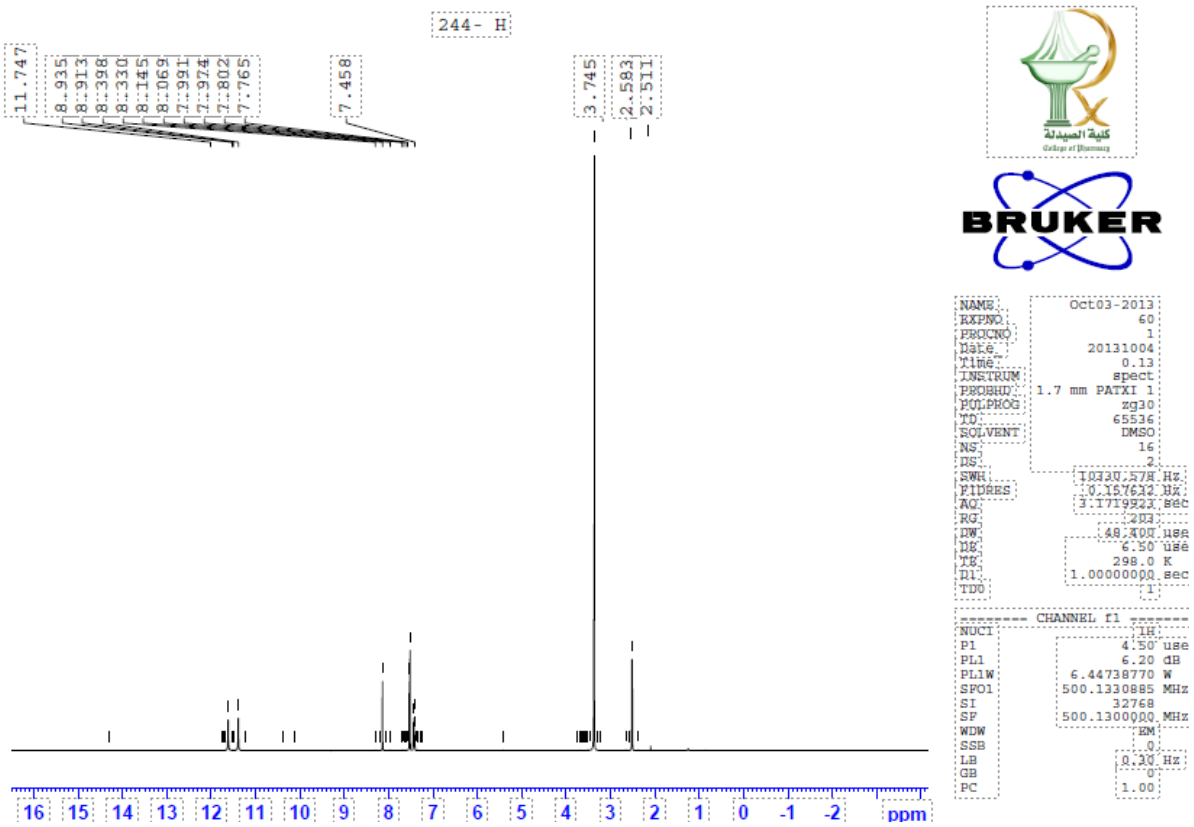
244- H
11.747
8.935
8.913
8.398
8.330
8.145
8.069
7.991
7.974
7.802
7.765
7.458
3.745
2.583
2.511
BRUKER
NAME Oct03-2013
EXPNO 60
PROCNO 1
Date_ 20131004
Time 0.13
INSTRUM spect
PROBHD 1.7 mm PATXI 1
PULPROG zg30
TD 65536
SOLVENT DMSO
NS 16
DS 2
SWH 10330.578 Hz
FIDRES 0.157632 Hz
AQ 3.1719923 sec
RG 203
DW 48.400 usec
DE 6.50 usec
TE 298.0 K
D1 1.00000000 sec
TD0 1
======== CHANNEL f1 ========
NUC1 1H
P1 4.50 usec
PL1 6.20 dB
PL1W 6.44738770 W
SFO1 500.1330885 MHz
SI 32768
SF 500.1300000 MHz
WDW EM
SSB 0
LB 0.30 Hz
GB 0
PC 1.00
16 15 14 13 12 11 10 9 8 7 6 5 4 3 2 1 0 -1 -2 ppm

3e

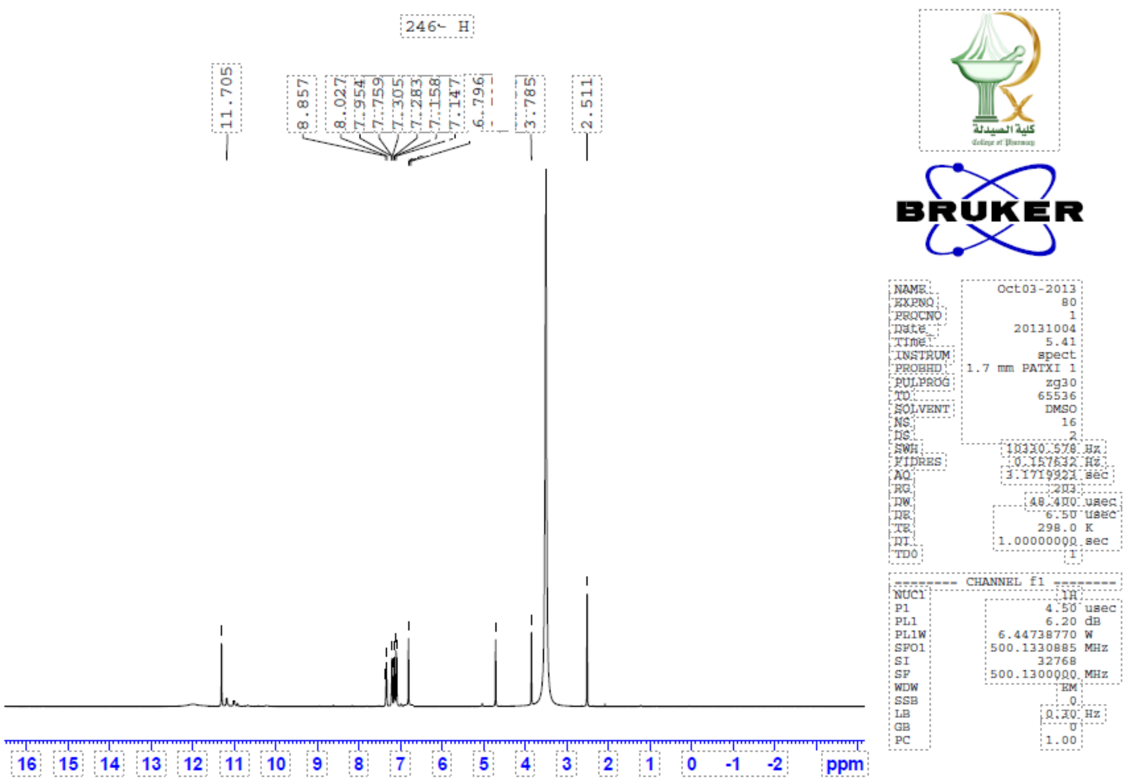

Fig. S2 NMR spectral analysis of the compounds 3a-3e: 3a (6-Iodo-4-(4-aminosulphonylphenyl-amino) quinazoline); 3b (6-Iodo-4-(4-aminosulphonylphenyl-amino)-2-(4-methoxyphenyl)-quinazoline); 3c (6-Iodo-2-phenyl-4-(4-(thiazol-2-yl) aminosulphonylphenyl-amino) quinazoline); 3d (6-Iodo-2-(4-methoxyphenyl)-4-(4-(thiazol-2-yl) aminosulphonylphenyl-amino)-quinazoline); and 3e (6-Iodo-2-(3,4-dimethoxyphenyl)-4-(4-(thiazol-2-yl) aminosulphonylphenyl-amino)-quinazoline).
